# Calpeptin attenuates LPS-induced ventriculomegaly in neonatal mice

**DOI:** 10.1016/j.bbrep.2026.102890

**Published:** 2026-11-10

**Authors:** Leandro Castañeyra-Ruiz, Jenna Ledbetter, Seunghyun Lee, Sora Sato, Gio Jison, Sydney Milo, Amirhossein Shahriari, Celine Thao-Quyen Tran, Alexandra Latini, Brian W. Hanak, Michael Muhonen

**Affiliations:** a CHOC Children's Research Institute, Orange, CA, 92868, USA; b Department of Pharmacology, University of La Laguna, La Laguna, 38071, Spain; c Division of Neurosurgery, CHOC Children's Hospital, Orange, CA, 92868, USA; d Department of Neurological Surgery, University of California Irvine, Orange, CA, 92868, USA

**Keywords:** Acquired hydrocephalus, Ventriculomegaly, TLR4, Calpain, Calpeptin, VE-Cadherin, Endothelial junctions, Neonatal inflammatory brain injury

## Abstract

Acute inflammatory insults in the developing brain can produce ventriculomegaly, but underlying mechanisms remain incompletely defined. We tested whether preventive calpeptin treatment modified the phenotype induced by intraventricular lipopolysaccharide (LPS) in neonatal mice. Ventricular-volume analysis included vehicle (12 pups/4 litters), LPS (8 pups/3 litters), and LPS + calpeptin (10 pups/2 litters). Calpeptin reduced mean ventricular volume by 72% relative to LPS (2.48 versus 8.92 mm^3^); the difference persisted in a mixed-effects model with litter as a random intercept (−0.61 log_10_ mm^3^, 95% CI −1.02 to −0.20, p = 0.004). Survival analysis included vehicle (13 pups/4 litters), LPS (26 pups/5 litters), and LPS + calpeptin (12 pups/2 litters). Observed endpoint survival was 10/12 with calpeptin versus 10/26 with LPS, but the litter-level comparison was not significant, and pronounced between-litter heterogeneity and only two calpeptin litters preclude a definitive treatment-effect inference; survival remains exploratory. Cortical DiI vascular labeling and VE-cadherin immunofluorescence were obtained from one litter per group and are descriptive, not measures of blood-brain barrier permeability. In cultured primary brain microvascular endothelial cells, LPS increased permeability and the approximately 95/130 kDa VE-cadherin-immunoreactive band ratio. Calpeptin and TLR4 inhibition reduced the band-ratio increase but did not significantly attenuate permeability relative to LPS. Calpeptin is not protease- or isoform-selective, and target engagement was not measured. Thus, the findings suggest a contribution from calpeptin-sensitive protease activity without identifying the responsible protease, confirming cleavage of the lower band, or demonstrating endothelial barrier protection in vivo.

## Highlights

• Intraventricular LPS produces ventriculomegaly in neonatal mice.
• Calpeptin reduced mean ventricular volume by 72% relative to LPS alone.
• Calpeptin-sensitive protease activity may contribute to pediatric hydrocephalus.

## 1 Introduction

Hydrocephalus is one of the most common and devastating neurological disorders of childhood, affecting infants worldwide and leading to lifelong motor, cognitive, and sensory impairments [1], [2], [3]. Despite its high prevalence and clinical burden, treatment remains largely surgical, relying on cerebrospinal fluid (CSF) diversion systems that are associated with high long-term failure and revision rates [4], [5], [6]. These limitations highlight the lack of effective disease-modifying therapies and underscore the need for a deeper mechanistic understanding of hydrocephalus pathogenesis.

Historically, hydrocephalus has been conceptualized as a disorder of CSF obstruction or impaired absorption, based on early experimental and clinical studies [7], [8], [9], [10], [11]. While these models established foundational principles of CSF dynamics, they do not adequately explain why similar insults lead to divergent outcomes, nor why hydrocephalus frequently progresses despite restoration of CSF flow. More recent work has emphasized the contribution of inflammation-driven injury to CSF-producing, CSF-contacting, and vascular interfaces of the developing brain [12], [13], [14]. Recent work has proposed that inflammatory injury in the preterm brain may interact with developmentally primed proteolytic systems within the germinal matrix vasculature, creating a vulnerability threshold during inflammatory exposure [15]. Together with evidence that ADAM10-dependent cleavage of N-cadherin disrupts the ventricular zone and produces ventriculomegaly after hemorrhage [16], this protease-threshold framework places junctional proteolysis at the interface between inflammatory exposure and ventricular enlargement [15], [16]. Whether calpeptin-sensitive protease activity contributes to inflammation-associated ventriculomegaly, and in which cellular compartment, remains unknown.

In particular, inflammatory hydrocephalus has emerged as a unifying framework linking post-hemorrhagic, post-infectious, and post-traumatic etiologies [12], [13]. Clinical and experimental studies have shown that innate immune activation disrupts choroid plexus function, alters CSF composition, and promotes ventricular enlargement [12], [13], [17]. However, the molecular mechanisms that translate inflammatory signaling into irreversible structural failure of brain barriers remain incompletely defined.

Emerging evidence indicates that disruption of cell-junction systems contributes to barrier failure in hydrocephalus. Disorganization of ventricular-zone adherens junctions has been associated with abnormal neurogenesis, white matter injury, and progressive ventriculomegaly [18], [19], [20], [21]. Experimental models further indicate that blood and inflammatory stimuli can disrupt the ventricular zone through junctional injury rather than mechanical obstruction alone [22], [23]. These findings raise the possibility that distinct insults activate proteases targeting specific cellular compartments while converging on cerebrospinal fluid dysregulation and hydrocephalus.

Proteolysis may therefore represent an effector mechanism connecting neuroinflammation to junctional failure. Metalloproteinases are induced in germinal-matrix and periventricular tissue after hemorrhagic and inflammatory insults, and loss of membrane-type 1 metalloproteinase impairs ependymal maturation and causes hydrocephalus in mice [24], [25], [26]. We recently showed that ADAM10 is upregulated in the disrupted ventricular zone of preterm neonates with intraventricular hemorrhage and in experimental posthemorrhagic hydrocephalus, where it cleaves N-cadherin [16]. ADAM10 activation reproduced ventricular-zone disruption and nonobstructive ventriculomegaly, whereas pretreatment with an ADAM10 inhibitor preserved N-cadherin and prevented ventricular enlargement [16]. These findings identify junctional proteolysis as a pharmacologically modifiable mechanism but leave unresolved whether an analogous process operates in the cerebral vasculature after a primarily inflammatory insult.

Toll-like receptor 4 (TLR4) is activated by pathogen- and damage-associated signals relevant to neonatal brain injury, including lipopolysaccharide and blood-breakdown products [27], [28]. TLR4 signaling can increase intracellular calcium and activate calpains, and published studies link calpain activity to VE-cadherin processing and endothelial permeability [29], [30], [31], [32], [33], [34], [35], [36], [37], [38], [39]. Whether pharmacological sensitivity to calpeptin is associated with inflammation-induced ventriculomegaly has not been tested. Because calpeptin is not protease- or isoform-selective, such an experiment cannot by itself distinguish calpains from cathepsins or establish that an endothelial pathway mediates ventricular enlargement.

We therefore tested whether preventive administration of calpeptin, initiated before and continued at the time of intraventricular LPS exposure, attenuates ventriculomegaly in neonatal mice. Complementary experiments in primary brain microvascular endothelial cells examined cultured-cell permeability and the relative abundance of approximately 95 and 130 kDa VE-cadherin-immunoreactive bands after LPS, calpeptin and TLR4-inhibitor exposure. The in vivo and in vitro experiments were interpreted as parallel pharmacological observations rather than as a demonstrated causal sequence.

## 2 Materials and methods

### 2.1 Ethics statement

All animal procedures were reviewed and approved by the Institutional Animal Care and Use Committee (IACUC) of CHOC Children's Hospital/CHOC Children's Research Institute, Orange, California, USA, under protocol number IACUC-24-004 (initial approval 13 August 2021; previously registered as protocol 210679). Magnetic resonance imaging was performed at the University of California, Irvine, under UC Irvine IACUC protocol AUP 20-172. All experiments were conducted in an AAALAC International–accredited facility, in compliance with the Animal Welfare Act, the U.S. Public Health Service Policy on Humane Care and Use of Laboratory Animals, and the Guide for the Care and Use of Laboratory Animals (8th edition). Reporting follows the ARRIVE 2.0 guidelines. Animals were monitored at least daily by trained personnel; animals showing excessive lethargy or muscle rigidity were reported to the attending veterinarian and treated or euthanized according to the approved humane endpoints in IACUC-24-004.

### 2.2 Study design, experimental units and group sizes

This study comprised one in vitro program and four in vivo experimental cohorts. Cohort 0 was a dose-selection (feasibility) experiment in which three litters received 2.5, 5.0 or 7.5 μg of intraventricular LPS (n = 5 pups per dose). Cohort 1 was an LPS-exposure experiment in which a single litter was divided between an LPS arm and a concurrent vehicle arm. Cohort 1 received the same 5-μg intraventricular LPS dose used in the subsequent modulation experiments. Cohorts 2 and 3 were two independent calpeptin-modulation experiments, each containing vehicle, 5-μg LPS and LPS + calpeptin arms. Treatment was allocated predominantly at the litter level: in eleven of the twelve litters used, every pup received the same intraventricular solution (Table 1). The exception is the cohort 1 litter, which was divided within the litter between LPS (n = 5) and vehicle (n = 2), and which therefore provides the only within-litter treatment contrast in the study. The in vivo design is accordingly a predominantly cluster-allocated design in which the litter is the unit of allocation and the pup is the unit of observation. Ten unique litters contributed to the pooled 5-μg treatment comparisons (four vehicle, five LPS and two LPS + calpeptin, with one litter shared by the vehicle and LPS arms); the two remaining litters were used only for 2.5- and 7.5-μg dose selection. Analyses model litter explicitly, and the effective sample size for treatment comparisons is closer to the ten contributing litters than to the number of pups. The experimental unit for all in vitro analyses is the independent culture well; each well contributed a single lysate loaded in a single lane, and wells were nested within two independent experiments performed on separate cell preparations.

**Table 1:** Complete animal accounting by litter for every in vivo group.

Cohort	Litter (dam)	Group	Injected	Died	Survived	Imaged (MRI)	Volumetry
0 — dose selection	Litter A (2.5 μg)	LPS 2.5 μg	5	0	5	2	2
0 — dose selection	LPS 1 (litter B)	LPS 5 μg	5	1	4	2	2
0 — dose selection	Litter C (7.5 μg)	LPS 7.5 μg	5	3	2	2	2
1 — LPS/vehicle	Vehicle 1 (split litter)	Vehicle	2	0	2	2	2
1 — LPS/vehicle	LPS 2 (split litter)	LPS 5 μg	5	5	0	0	0
2 — modulation 1	Vehicle 2 (control #1)	Vehicle	3	1	2	2	2
2 — modulation 1	Vehicle 3 (control #2)	Vehicle	2	0	2	2	2
2 — modulation 1	LPS 3	LPS 5 μg	6	5	1	1	1
2 — modulation 1	Calpeptin 1	LPS + calpeptin	5	1	4	4	4
3 — modulation 2	Vehicle 4	Vehicle	6	0	6	6	6
3 — modulation 2	LPS 4	LPS 5 μg	5	0	5	5	5
3 — modulation 2	LPS 5	LPS 5 μg	5	5	0	0	0
3 — modulation 2	Calpeptin 2	LPS + calpeptin	7	1	6	6	6
Pooled	4 litters	Vehicle	13	1	12 (92.3%)	12	12
Pooled	5 litters	LPS	26	16	10 (38.5%)	8	8
Pooled	2 litters	LPS + calpeptin	12	2	10 (83.3%)	10	10

Exact numbers of animals allocated, surviving, imaged and analyzed are given for every group in Table 1 and Supplementary Tables S1–S4. Table 2 summarizes the observations, independent litters or experiments, statistical unit and principal limitation for every quantitative endpoint. Ventricular volume was measured only in animals surviving to endpoint MRI and is therefore conditional on survival.

**Table 2:** Experimental replication, statistical unit and interpretation for each quantitative endpoint.

Endpoint	Observations	Independent structure	Statistical unit/analysis	Interpretive limit
Survival	Vehicle 13; LPS 26; LPS + calpeptin 12 pups	4, 5 and 2 litters; one split litter	Pup outcome; litter-aware sensitivity analyses	Exploratory; only 2 calpeptin litters and marked LPS litter heterogeneity
Ventricular volume	12; 8; 10 surviving imaged pups	4, 3 and 2 litters	Pup; mixed model with litter random intercept	Primary in vivo result; conditional on survival
Body weight	6; 5; 6 surviving pups	1 litter per group	Repeated animal observations; descriptive	Treatment, litter and survival are confounded; no pretreatment baseline
DiI labeling	6/12; 5/9; 5/9 animals/hemispheres	1 litter per group	Animal with paired hemispheres; descriptive	Treatment and litter are confounded; DiI is not permeability
VE-cadherin IF	6; 4; 4 animals	1 litter per group	Animal-level mean; descriptive	Treatment and litter are confounded
Permeability	6; 4; 6; 6 wells	1 plate/experiment	Independent culture well; Kruskal-Wallis/Dunn-Holm	Single-experiment cultured-cell result
VE-cadherin immunoblot	9; 9; 9; 7 wells	2 cell preparations; 3 membranes	Independent well; experiment blocking factor	No useable loading control; experiment 2 treatment partly confounded with membrane
Dose selection	5 pups per LPS dose	1 litter per dose	Descriptive	Feasibility study; not a powered dose-response

### 2.3 Animals and treatment allocation

Wild-type C57BL/6 mice were obtained from The Jackson Laboratory and bred at the CHOC animal facility. Animals were housed in a temperature-controlled room (22 °C) under a 12 h light/12 h dark cycle with standard chow and water available ad libitum. Pups of both sexes were used. Injections were performed on postnatal day 3, at which age sex cannot be reliably determined without genotyping, and the sex of individual animals was not recorded; sex could therefore not be included as a biological variable. Because litters were allocated whole, both sexes are expected to be represented in every treatment arm in approximately the proportions occurring naturally within a litter. Twelve litters, from twelve separate dams, were used across the four in vivo cohorts. Ten contributed to the pooled 5-μg treatment comparisons; the other two received 2.5 or 7.5 μg LPS only in the dose-selection experiment. Of the ten pooled-comparison litters, nine were allocated entirely to one treatment: three vehicle litters (two in calpeptin-modulation experiment 1, one in experiment 2), four LPS litters (one in cohort 0, one in experiment 1, and two in experiment 2), designated LPS 1 and LPS 3–LPS 5 in Fig. 2D and E, and two LPS + calpeptin litters (one per modulation experiment). The tenth pooled-comparison litter, in cohort 1, was divided within the litter between LPS (n = 5) and vehicle (n = 2). Litter membership for every animal is given in Tables 1 and in Supplementary Table S1–S4. Because allocation was predominantly at litter level, treatment effects are estimated largely between litters rather than within them, and all in vivo analyses either model litter as a random effect, use litter-level cluster-robust inference, or are presented at the level of the litter (Section 2.13). No formal a priori power calculation was performed; sample sizes were based on prior intraventricular inflammation studies. Investigators performing MRI segmentation and image quantification were not blinded to treatment allocation; this is acknowledged as a limitation.

### 2.4 Induction of intraventricular inflammation

For the dose-selection experiment, three groups of P3 mice (n = 5 analysable pups per group) received intraventricular injections of lipopolysaccharide (LPS; Sigma-Aldrich, #L3024; *E. coli* O111:B4) at volumes of 1, 2 or 3 μL of a 2.5 μg/μL stock, corresponding to total doses of 2.5, 5.0 and 7.5 μg, prepared in vehicle consisting of 5% glycerol, 85% PBS (pH 7.4) and 10% DMSO.

For the calpeptin-modulation experiments, litters were allocated to three groups receiving 2 μL intraventricular injections of: (i) vehicle (5% glycerol, 85% PBS, 10% DMSO); (ii) 5 μg LPS in vehicle; or (iii) 5 μg LPS combined with 15 μg calpeptin (Sigma-Aldrich, #C8999) in the same vehicle. Final intraventricular concentrations were 2.5 μg/μL LPS and 7.5 μg/μL calpeptin (≈20 mM calpeptin in the injectate; molecular weight 362.5 g/mol).

Injections were performed in the right lateral ventricle at P3 using stereotactic coordinates (0.8 mm lateral, 1.7 mm anterior, 1.5 mm depth from the posterior fontanelle). Solutions were delivered over 30 s using a 25 μL Hamilton syringe (80366) mounted on a UMP3 Ultra Micropump (World Precision Instruments). Neonatal mice were anesthetized by hypothermia during the procedure.

In addition to the intraventricular injection, animals in the LPS + calpeptin group received subcutaneous calpeptin (75 μg in 10 μL of 20% DMSO/80% PBS) 24 h and 3 h before the intraventricular procedure. Vehicle and LPS animals did not receive matched subcutaneous injections. The absence of an injection-matched subcutaneous vehicle control is acknowledged as a design limitation (Section 4.6); consequently, a contribution of the subcutaneous procedure or of subcutaneous DMSO to the observed differences cannot be formally excluded. No systematic scoring of injection-site irritation was performed, and local vehicle-related effects therefore cannot be excluded.

### 2.5 Body weight and clinical monitoring

Pups were weighed on a digital balance at pre-specified intervals from the day after injection until the endpoint (Supplementary Table S1–S4). Animals were inspected at least once daily for lethargy, muscle rigidity, failure to nurse and cutaneous lesions; observations were recorded in the animal facility records. Mortality was recorded as the date on which an animal was found deceased during a scheduled check. Because animals were inspected at fixed intervals rather than continuously, the recorded time of death is the time of discovery and the true time of death lies within the preceding inspection interval (interval censoring of up to approximately 24–48 h); survival times should be read accordingly. Six non-injected littermates were maintained alongside cohort 0 as an untreated growth reference and are reported in Supplementary Table S1; they were not part of any treatment comparison and are excluded from all inferential analyses. Because clinical signs were recorded qualitatively rather than by a validated ordinal severity score, only body weight and mortality are analyzed quantitatively.

### 2.6 Magnetic resonance imaging

Fourteen days after induction of neuroinflammation, mice underwent in vivo MRI at the UC Irvine Preclinical and Translational Neuroimaging Center. Imaging was performed using a 4.7 T horizontal-bore Agilent MRI system equipped with a 2 cm inner-diameter quadrature RF coil. Animals were anesthetized with isoflurane and maintained under controlled airflow with continuous monitoring of respiration and body temperature. T2-weighted images were acquired and telencephalic ventricular volumes were segmented manually using ITK-SNAP, as previously described [40]. Only animals alive at the imaging time point could be scanned; ventricular volumes are therefore reported for survivors only, with the corresponding denominators given in Table 1. In addition, not every survivor was imaged: in cohort 0, scanner availability limited imaging to two animals per dose, and these were selected in tattoo order rather than at random. Imaged animals are therefore a non-random subset of survivors, which is stated as a limitation in Section 4.6.

### 2.7 Vessel labeling and vascular imaging

Following MRI, mice were euthanized and perfused transcardially with PBS (150 mL), then with the lipophilic carbocyanine dye DiI (Invitrogen, #D282; 50 mL at 13 μg/mL), and finally with 4% paraformaldehyde (200 mL). Brains were extracted and cortical vasculature was imaged using a Keyence BZ-X810 fluorescence microscope. DiI fluorescence intensity was quantified in ImageJ within a region of interest in the cortex dorsal to the lateral ventricle, with background subtraction. Both hemispheres were measured in each animal and hemisphere side was retained in the descriptive summaries. Two hemispheres, one in the LPS group and one in the LPS + calpeptin group, were excluded because the tissue was artifactually damaged at the site of measurement. The analyzed animals came from cohort 3 and represented one surviving litter per treatment group (vehicle n = 6, LPS n = 5 and LPS + calpeptin n = 5); the second LPS litter in that cohort had no survivors. Treatment and litter are therefore completely confounded for this endpoint. DiI reports vascular labeling and perfusion, not blood-brain barrier permeability or tracer extravasation.

### 2.8 Immunofluorescence

Brain tissue was fixed, dehydrated and embedded in paraffin. Sections (10 μm) were deparaffinized using xylene and graded ethanol washes. Antigen retrieval was performed in citrate buffer (pH 6.0) at 100 °C for 20 min. Sections were incubated with anti-VE-cadherin antibody (Invitrogen, #PA5-19612; 1:25) overnight, followed by Alexa Fluor 488-conjugated anti-rabbit antibody (Thermo Fisher, #A11034; 1:400) for 2 h. Slides were mounted with Fluoromount-G containing DAPI and imaged on an ECHO Revolve fluorescence microscope. Multiple fields were measured per animal and averaged to one animal-level value. The analyzed animals came from one cohort 3 litter per treatment group (vehicle n = 6, LPS n = 4 and LPS + calpeptin n = 4), so these data are reported descriptively and cannot distinguish treatment from litter.

### 2.9 Primary brain endothelial cell culture

Primary C57BL/6 mouse brain microvascular endothelial cells were obtained from Cell Biologics (catalog C57-6023). According to the manufacturer's product documentation, the cells are supplied cryopreserved at passage 3, are characterized using endothelial markers including VE-cadherin and CD31/PECAM-1 or DiI-acetylated-low-density-lipoprotein uptake, and are screened for bacteria, yeast, fungi and mycoplasma. No additional identity authentication or mycoplasma testing was performed in our laboratory after receipt. Endothelial phenotype in the cultures used here was supported by cobblestone morphology and VE-cadherin immunofluorescence (Fig. 1A). The supplier does not specify donor age, developmental stage or sex; these cells are therefore not characterized as a neonatal endothelial model, and developmental equivalence to P3 brain endothelium was not established. Cells were used at passages 3–5 and cultured in complete mouse endothelial cell medium (Cell Biologics, kit M1168) on gelatin-coated vessels at 37 °C and 5% CO_2_, with medium refreshed every 48 h. For permeability experiments, cells were seeded at 5 × 10^4^ cells/cm^2^ onto polyester Transwell inserts (0.4 μm pore size) and used after formation of a continuous confluent monolayer. Cells for protein extraction were grown to confluence in parallel multiwell plates.

**Fig. 1:**
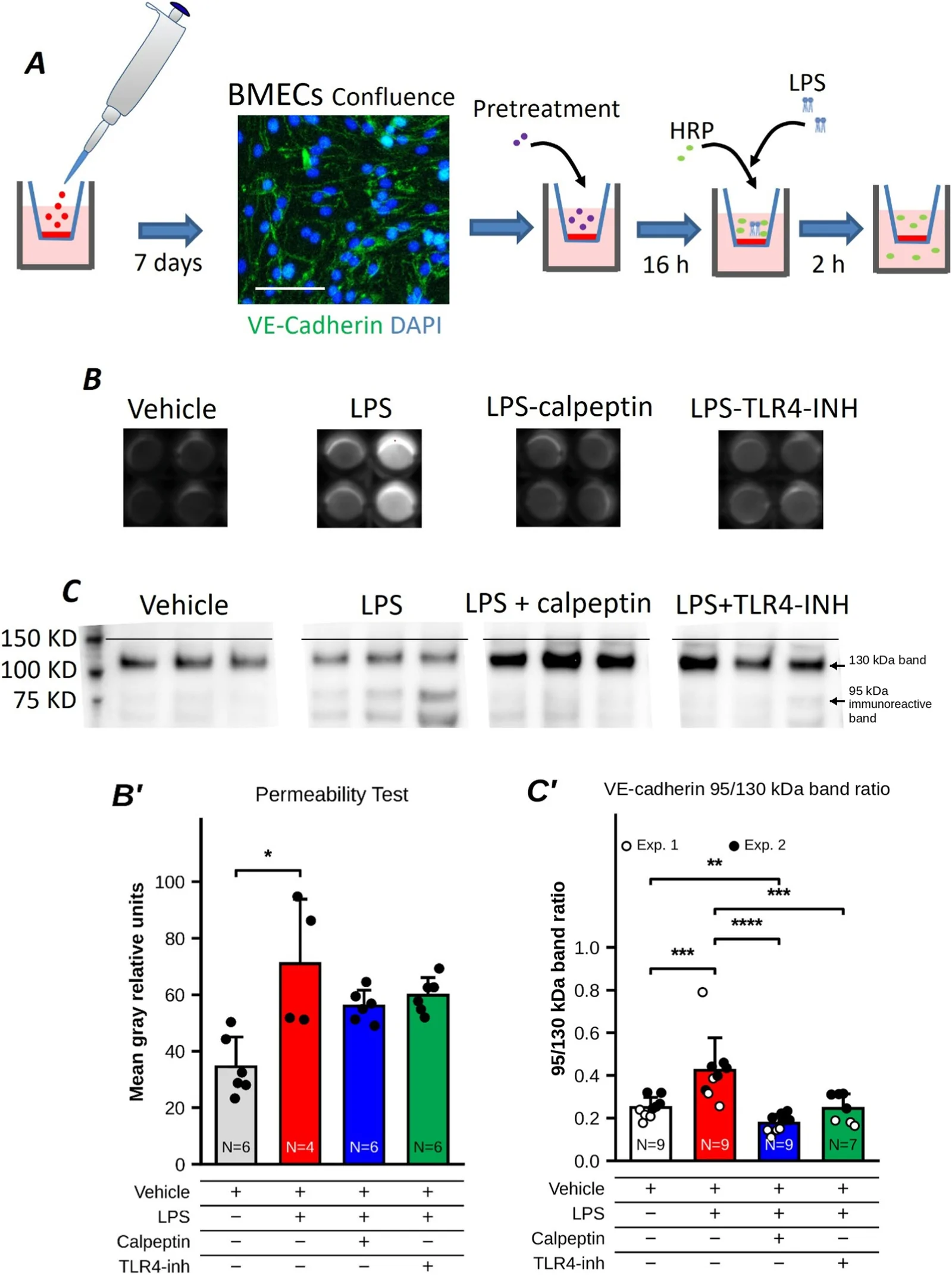
LPS increases endothelial permeability and the approximately 95/130 kDa VE-cadherin-immunoreactive band ratio in cultured brain microvascular endothelial cells. (A) Primary brain microvascular endothelial cells were grown to confluence, pretreated with vehicle, calpeptin or TLR4 inhibitor for 16 h, and then exposed to LPS with streptavidin-HRP for 2 h while the corresponding inhibitor remained present. Scale bar, 50 μm. (B,B′) Representative permeability readout and quantification from one experiment (vehicle n = 6 wells, LPS n = 4, LPS + calpeptin n = 6, LPS + TLR4 inhibitor n = 6). Bars show mean + SD and individual wells. LPS exceeded vehicle after Dunn-Holm adjustment (p = 0.029); neither inhibitor condition differed significantly from LPS alone. (C) Representative VE-cadherin immunoblot with molecular-weight markers and neutral on-panel annotations identifying the “130 kDa band” and the “95 kDa immunoreactive band”; the lower band has not been identified as a cleavage product. (C′) Within-lane approximately 95/130 kDa VE-cadherin-immunoreactive band ratio (not a confirmed fragment/full-length cleavage ratio) from two independent cell preparations; each point is one independent well quantified once (vehicle n = 9, LPS n = 9, LPS + calpeptin n = 9, LPS + TLR4 inhibitor n = 7). Open and filled symbols indicate experiments 1 and 2. Bars show mean + SD. The lower-molecular-weight band was not independently identified as a cleavage fragment. Full statistics are reported in Supplementary Table S5. Statistical symbols: in B′, * denotes p = 0.029 (Dunn–Holm comparison after the Kruskal–Wallis test); in C′, ** denotes p = 0.0055, *** denotes p = 0.0001, and **** denotes p < 0.0001 (Tukey-adjusted comparisons following ANOVA of log_10_ band ratios with experiment as a blocking factor). Symbols refer to the indicated pairwise brackets; full results appear in Supplementary Table S5.

**Fig. 2:**
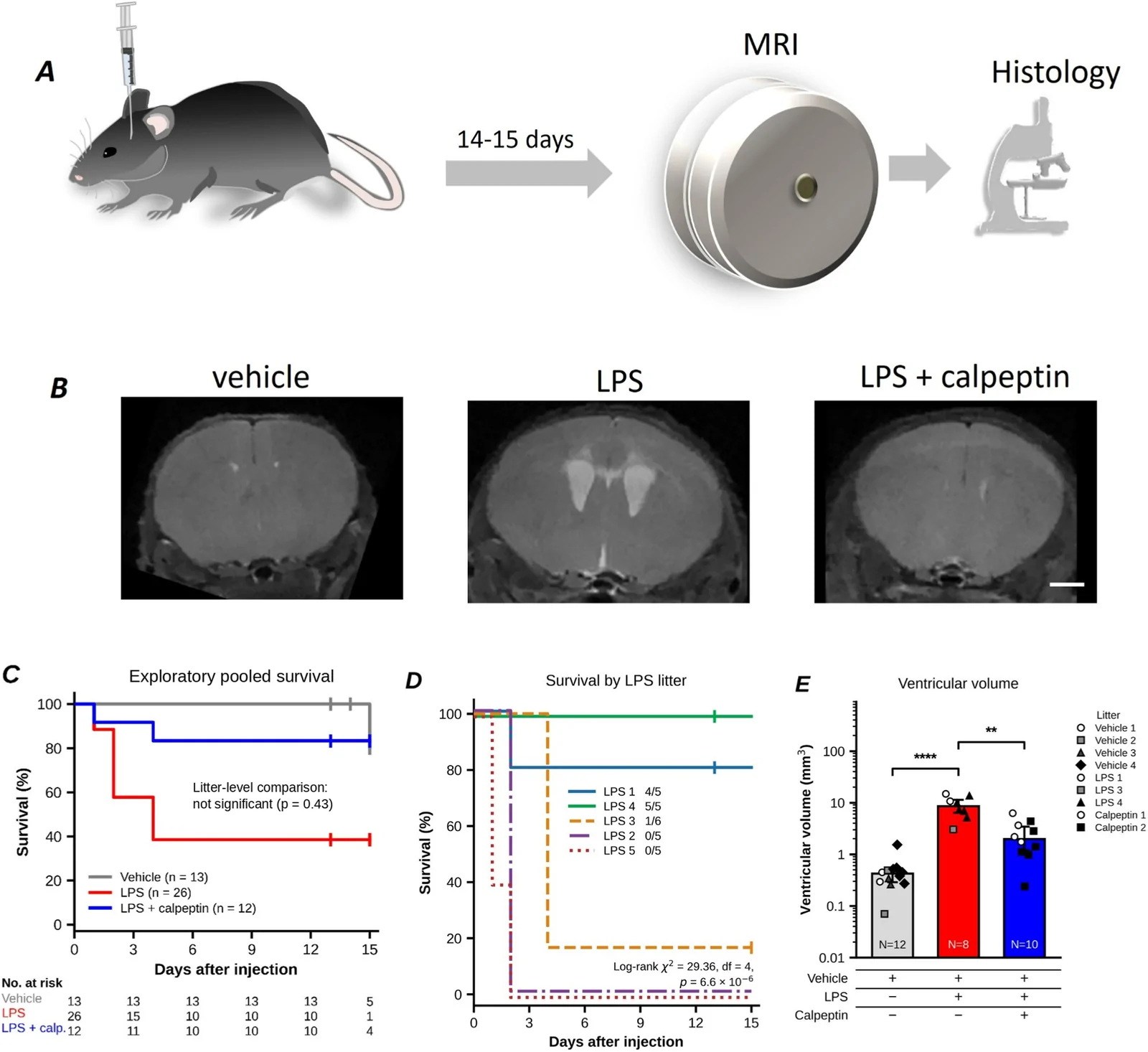
Calpeptin attenuates ventriculomegaly after intraventricular LPS; survival differences are exploratory. (A) Pups received intraventricular injections at P3 and underwent MRI 14-15 days later. (B) Representative coronal T2-weighted MRI images. Scale bar, 2 mm. (C) Exploratory pooled survival: Kaplan-Meier curves with numbers at risk (vehicle 13 pups/4 litters; LPS 26/5; LPS + calpeptin 12/2). The pooled endpoint survival proportion was higher with calpeptin than LPS alone, but this observation is exploratory, not evidence of a confirmed treatment effect: the litter-level LPS versus LPS + calpeptin comparison was not significant (p = 0.43; five versus two litters), and survival varied markedly between LPS litters. (D) Survival curves for individual LPS litters illustrate marked between-litter heterogeneity. (E) Ventricular volumes in surviving imaged animals (vehicle 12 pups/4 litters; LPS 8/3; LPS + calpeptin 10/2). The primary log10 mixed-effects model included a random intercept for litter; LPS + calpeptin differed from LPS (p = 0.004). Volumes are conditional on survival. Full statistical reporting is provided in Supplementary Table S5. Statistical brackets in (E): **** denotes vehicle versus LPS (p < 0.0001, Tukey-adjusted comparison following ANOVA of log_10_ vol; secondary analysis), whereas ** denotes LPS versus LPS + calpeptin (p = 0.004, primary mixed-effects model with a random litter intercept). The primary model also supported the vehicle-versus-LPS comparison (p < 0.001).

For the Western blot experiments, two independent experiments were performed on separate cell preparations and resolved on three membranes. Experiment 1 used one membrane containing vehicle, LPS and LPS + calpeptin (four wells each) and LPS + TLR4 inhibitor (three wells). Experiment 2 used one membrane containing vehicle and LPS (five wells each) and a second membrane containing LPS + calpeptin (five wells) and LPS + TLR4 inhibitor (four wells). Each independent culture well contributed one lysate, one lane and one quantified value; no technical replicate was counted as an independent observation. The approximately 95/130 kDa band ratio was calculated within each lane, and experiment was retained as a blocking factor. Because the inhibitor groups in experiment 2 were resolved on a different membrane from vehicle and LPS, membrane and treatment cannot be separated fully in that experiment; experiment 1 provides the complete within-membrane comparison. The permeability experiment was performed once on one plate and is reported as a single-experiment result. Uncropped membranes from both experiments are provided in the Supplementary Material.

Cells assigned to inhibitor conditions were pretreated with calpeptin or the TLR4 inhibitor for 16 h. At assay initiation, LPS and streptavidin-HRP were added and the corresponding inhibitor was also present during the subsequent 2-h exposure. Final concentrations during the assay were LPS 5 μg/mL, calpeptin 7.5 μg/mL (approximately 20.7 μmol/L), TLR4 inhibitor 1 μg/mL and vehicle 5% glycerol/85% PBS/10% DMSO. Residual DMSO was matched across conditions. This preventive schedule does not test reversal after an established permeability change.

### 2.10 Pharmacological agents

Calpeptin (Z-Leu-Nle-CHO; Sigma-Aldrich #C8999) is a cell-permeable peptide aldehyde originally developed as a calpain inhibitor [41]. It is not calpain-selective: it inhibits calpain-1 and calpain-2 without isoform discrimination and, as recently established by enzyme kinetics and X-ray crystallography, inhibits cysteine cathepsins K and L with picomolar inhibition constants (Ki 61 pM and 131 pM respectively), i.e. with potency equal to or greater than its activity against calpains [42]. Calpeptin is therefore used here as a broad-spectrum cysteine protease inhibitor. Target engagement was not directly measured; no calpain activity assay, calpain-specific substrate cleavage assay or cathepsin activity assay was performed. Accordingly, no result in this study can be attributed to calpain-1, to calpain-2, or to calpains as opposed to cathepsins or other cysteine proteases.

The TLR4 inhibitor used in vitro was Novus Biologicals #NBP2-26244, applied at a final concentration of 1 μg/mL. No inactive control peptide was included in the present experiments. TLR4 inhibition was not verified with a downstream readout: NF-κB nuclear translocation, IκBα degradation and cytokine release (TNF-α, IL-1β, IL-6) were not measured. The in vitro TLR4 inhibitor result is therefore reported as pharmacological evidence consistent with TLR4 involvement, and not as demonstration of TLR4 dependence.

### 2.11 Western blot

Cells were lysed in RIPA buffer (Thermo Fisher, #89900) supplemented with Laemmli sample buffer and reducing agent and heated at 70 °C for 10 min. Protein samples (20 μL per lane) were resolved by SDS-PAGE and transferred using Trans-Blot Turbo transfer packs (Bio-Rad, #1704158). Membranes were blocked in nonfat milk in PBS for 45 min and incubated overnight at 4 °C with anti-VE-cadherin (1:250; Invitrogen, #PA5-19612), followed by goat anti-rabbit secondary antibody (Thermo Fisher, #A11034; 1:10,000) for 2 h at room temperature. GAPDH immunoblotting (Abcam, #ab8245) was attempted, but the resulting signal was technically uninterpretable and was not used for analysis. The prespecified endpoint was the within-lane ratio between the two VE-cadherin-immunoreactive bands, not total VE-cadherin abundance.

Molecular weights were assigned using a co-migrating pre-stained ladder visible in the uncropped membranes. Bands were quantified in ImageJ using fixed-area regions of identical size with local background subtraction. The approximately 130 kDa band was considered consistent with glycosylated full-length VE-cadherin, which migrates above its approximately 88 kDa polypeptide mass because of extensive N-glycosylation [43], whereas the approximately 95 kDa signal was designated a lower-molecular-weight VE-cadherin-immunoreactive band. Its identity was not confirmed by mass spectrometry, N-terminal sequencing or a cleavage-site-specific antibody. Molecular weight alone cannot establish cleavage, intracellular localization or biological significance; the signal could represent a cleavage or degradation product, an altered isoform, a post-translationally modified species, nonspecific immunoreactivity or another form of protein processing. For each lane, the approximately 95/130 kDa signal ratio was calculated. A multiplicative lane-loading factor common to both bands cancels in this within-lane ratio, but the absence of a useable loading control precludes conclusions about absolute or total VE-cadherin abundance.

### 2.12 Endothelial permeability assay

Streptavidin–horseradish peroxidase (HRP) was added to the upper chamber at a final concentration of 0.1 μg/mL (1 μg in 10 mL) and incubated for 2 h. Following treatment, 50 μL of medium from the lower chamber of each well was collected and mixed with 50 μL SuperSignal West Pico Plus chemiluminescent substrate (Thermo Fisher, #34577). Chemiluminescence was measured using a ChemiDoc imaging system and quantified as integrated density in ImageJ. Each well was sampled once and read once; no well contributed more than one value to the analysis. Two assay controls were included on the same plate: cell-free inserts, which define the maximum attainable tracer transfer in the absence of a monolayer, and wells to which no streptavidin–HRP was added, which define the background signal. The cell-free control measured 104.9 integrated density units and the no-HRP wells were at background. Because a well cannot present less resistance to tracer transfer than a cell-free insert, any well whose signal exceeded the cell-free control was taken to indicate failure of monolayer confluence rather than a measurement of barrier function, and was excluded before analysis. This criterion was defined by the plate controls, was applied without reference to treatment group, and excluded four of twenty-six wells (two vehicle and two LPS; values 126.1, 138.9, 188.4 and 201.3). All twenty-two retained wells fell below the cell-free control, the highest being 94.8. The excluded wells and the effect of the exclusion on the rank-based comparison are described in Supplementary Table S5.

### 2.13 Statistical analysis

All analyses were performed in GraphPad Prism 10 and in Python 3.12 (lifelines 0.30.3, statsmodels, SciPy). Individual data points are shown in every quantitative graph and exact group sizes are given in every figure legend.

Survival was analyzed as time to death using Kaplan–Meier estimation, with surviving animals censored on the terminal day of their cohort (day 13, 14 or 15 after injection). Curves are displayed with censoring ticks and numbers at risk. Because allocation was predominantly at litter level, heterogeneity between litters was assessed before any treatment comparison, by log-rank comparison of the five LPS litters and, separately, of the litters within each of the other two arms. Survival is then reported at three levels of analysis, all of which are presented rather than only the most favorable: (i) a pup-level Cox proportional-hazards model stratified by cohort, which ignores within-litter correlation and therefore yields the narrowest intervals; (ii) the same model with cluster-robust standard errors by litter, noting that robust variance estimation with only ten clusters is anti-conservative and that the resulting intervals should not be read as more reliable than the pup-level ones; and (iii) a litter-level analysis in which the proportion surviving within each litter is the observation, which respects the unit of allocation but has very low power with two to five litters per arm. The within-litter contrast available in cohort 1 was analyzed by Fisher exact test. Proportional hazards were assessed by scaled Schoenfeld residuals. Sensitivity analyses comprised an unstratified pairwise log-rank test with Holm correction, a within-cohort permutation test (2000 permutations) of the stratified Cox z statistic, a ridge-penalised Cox model, and a Fisher exact test on the pooled proportions. Standard maximum-likelihood estimation converged with finite confidence intervals, so a Firth-penalised model was not required. All survival treatment comparisons are exploratory, because allocation was predominantly by litter, only two calpeptin-treated litters were available, and substantial LPS-litter heterogeneity was observed. Pup-level p values do not establish a treatment effect at the litter allocation level.

Ventricular volume. Group standard deviations differed by more than an order of magnitude, so analysis on the raw scale violates the equal-variance assumption of one-way ANOVA (Brown–Forsythe and Bartlett tests both p < 0.0001). Volumes were therefore log_10_-transformed, which restored variance homogeneity (Levene p = 0.45). The primary analysis was a linear mixed-effects model of log_10_ vol with treatment as a fixed effect and a random intercept for litter, which accounts for the cluster allocation described in Section 2.2. Analyses that make no equal-variance assumption — a Kruskal–Wallis test with Dunn post hoc comparisons and Holm adjustment, and Games–Howell comparisons on the raw scale — and a one-way ANOVA with Tukey post hoc comparisons on log_10_ vol are reported alongside it, together with litter-level means and the intraclass correlation attributable to litter.

Body weight. Serial weights in cohort 3 are presented descriptively. Each treatment arm consisted of one litter, treatment and litter were therefore completely confounded, and no pre-injection baseline weight was available. Repeated measurements within an animal do not provide independent treatment replication. Accordingly, no inferential treatment-effect test is used and weight is not interpreted as evidence of neurological or functional recovery.

Immunofluorescence and in vitro assays. In vivo VE-cadherin immunofluorescence is summarized descriptively at the animal level because each treatment group came from one litter. For the in vitro assays, the independent culture well is the statistical unit. One-way ANOVA with Tukey-adjusted comparisons was used for the Western-blot ratios after log10 transformation and inclusion of experiment as a blocking factor; Kruskal-Wallis testing with Dunn-Holm comparisons was used for the single permeability experiment. Exact group sizes and individual observations are shown.

Vessel labeling. Each animal contributed up to two hemisphere measurements, which are paired rather than independent. Hemisphere values are displayed with measurements from the same animal joined, and per-animal summaries are reported. Because the vehicle, surviving LPS and LPS + calpeptin animals each came from a single litter, treatment and litter are completely confounded and no inferential treatment comparison is made for this endpoint.

The dose-selection experiment is analyzed descriptively.

Injected = number of pups that received an intraventricular injection. Died = animals found deceased before endpoint MRI. Imaged = survivors that were successfully scanned. Volumetry = animals contributing a ventricular volume to Fig. 2E. Ventricular volume was measured only in survivors and is therefore conditional on survival. Each of the twelve litters came from a separate dam; ten contributed to the pooled 5-μg comparisons. In eleven of the twelve litters every pup received the same treatment, so the litter is the unit of allocation; the cohort 1 litter is the single exception, being divided within the litter between LPS (n = 5) and vehicle (n = 2), and it provides the only within-litter treatment contrast in the study. The cohort 1 LPS arm received 5 μg LPS. Litters LPS 4 and LPS 5 were both injected with 5 μg LPS on the same day within calpeptin-modulation experiment 2, yet all five pups of LPS 4 survived and all five pups of LPS 5 died. Cohort 1 animals contribute to the pooled LPS and vehicle arms but received no calpeptin. Litters are designated by treatment and number as used in Fig. 2D and E, with the original colony identifier in parentheses where it differs; the 2.5-μg and 7.5-μg dose-selection litters are not part of the pooled LPS arm and are therefore not numbered. Only the 5.0-μg litter of cohort 0 contributes to the pooled LPS arm from the dose-selection cohort; the 2.5-μg and 7.5-μg litters of cohort 0 are shown for completeness and are analyzed only in Section 3.3 and Fig. S1.

Table 2 distinguishes endpoints replicated across independent litters or cell preparations from descriptive endpoints obtained from one litter per treatment group.

## 3 Results

### 3.1 LPS increases endothelial permeability in vitro; calpeptin and TLR4-inhibitor conditions were intermediate but did not differ from LPS alone

Confluent primary brain microvascular endothelial cells were pretreated for 16 h and then exposed to LPS with or without calpeptin or a TLR4 inhibitor during the 2-h streptavidin-HRP assay. LPS approximately doubled tracer transfer relative to vehicle (median 69.0 versus 30.6; mean 71.0 versus 34.5 integrated density units; Fig. 1A,B,B'). Across the four conditions the difference was significant (Kruskal-Wallis H = 12.60, p = 0.0056), with LPS higher than vehicle after Dunn-Holm adjustment (p = 0.029). Calpeptin and TLR4-inhibitor conditions showed intermediate values, corresponding to removal of 41% and 31% of the observed LPS-associated increment, but neither differed significantly from LPS alone and the TLR4-inhibitor condition remained above vehicle. Thus, any attenuation of the permeability increase was directional only and was not statistically supported in this single culture experiment. The experiment does not identify a responsible protease, establish calpain dependence or demonstrate endothelial barrier protection in vivo.

### 3.2 LPS increases a lower-molecular-weight VE-cadherin–immunoreactive band, and calpeptin and TLR4 inhibition reduce the increase

Western blotting showed that LPS increased the within-lane ratio of the approximately 95 kDa to approximately 130 kDa VE-cadherin-immunoreactive bands by 70% relative to vehicle (mean 0.424 versus 0.249; Fig. 1C–C'). Ratios were log10-transformed and analyzed with experiment as a blocking factor because the two independent experiments differed in overall ratio (block effect p = 0.0003). The treatment effect was significant (F(3,29) = 24.86, p < 0.0001), and within-experiment sensitivity analyses were concordant in direction. In Tukey-adjusted comparisons, LPS exceeded vehicle (p = 0.0001), and the ratios in the calpeptin and TLR4-inhibitor conditions were lower than LPS alone (p < 0.0001 and p = 0.0001). Full statistical reporting is provided in Supplementary Table S5.

The approximately 95 kDa band is not assigned as a confirmed cleavage fragment. Its migration overlaps lower-molecular-weight VE-cadherin species reported after protein processing in other endothelial injury models [44], [45], but apparent molecular weight alone cannot identify the biochemical event, cellular compartment or responsible protease. The band could reflect a cleavage or degradation product, an altered isoform, a post-translationally modified species, nonspecific immunoreactivity or another form of protein processing. Its identity will require orthogonal validation.

Calpeptin and the TLR4 inhibitor produced parallel changes in the band ratio and permeability assay, but these endpoints were not linked experimentally at the level of individual wells and no mediation experiment was performed. The lower-molecular-weight band is therefore considered a candidate correlate of the pharmacological response rather than a validated mediator of permeability.

### 3.3 Dose selection for the intraventricular LPS model

A small feasibility experiment was performed to select an intraventricular LPS dose for the subsequent pharmacological experiments (Fig. S1, Table S1). Five pups received each of three doses. At 2.5 μg all five animals survived and the two animals that were imaged had ventricular volumes of 5.80 and 8.48 mm^3^. At 5.0 μg, four of five animals survived and the two imaged animals had volumes of 14.84 and 10.74 mm^3^. At 7.5 μg, three of five animals died within 2 days of injection and the two imaged survivors had volumes of 24.74 and 22.13 mm^3^.

The 5 μg dose was selected as a pragmatic compromise because it produced detectable ventriculomegaly while retaining sufficient survival for endpoint imaging and avoiding the higher mortality observed at 7.5 μg. This experiment was not powered to establish a quantitative dose–response relationship, and 5 μg is not described as an optimal dose; all individual observations are shown in Fig. S1 and Table S1. Published neonatal rodent studies have used a wide range of intracerebral and intraventricular LPS doses; because those protocols differ in species, age, route and endotoxin serotype, they support the general feasibility of this model but cannot be used to validate the specific dose used here, and systemic LPS studies are not informative for an intracerebral dose.

### 3.4 Calpeptin attenuates LPS-induced ventriculomegaly

Ventricular volumes were measured at endpoint MRI in all surviving animals that could be imaged (vehicle n = 12; LPS n = 8; LPS + calpeptin n = 10; Table 1). LPS produced marked ventriculomegaly (mean 8.92 ± 4.10 mm^3^, median 8.57, range 3.03–14.84) compared with vehicle (mean 0.47 ± 0.36 mm^3^, median 0.42, range 0.07–1.53), a 19.0-fold increase in the mean. Calpeptin reduced ventricular volume to 2.48 ± 1.84 mm^3^ (median 1.97, range 0.24–6.27), a 72% reduction in mean ventricular volume relative to LPS alone.

Group standard deviations differed by more than an order of magnitude (0.36, 4.10 and 1.84 mm^3^ for vehicle, LPS and LPS + calpeptin respectively), and volumes were therefore log_10_-transformed, which restored variance homogeneity (Levene p = 0.45). A distribution-free Kruskal–Wallis test with Dunn–Holm comparisons gave the same ordering of groups (H = 21.01, p = 2.7 × 10^−5^; pairwise p = 2.0 × 10^−5^, 0.038 and 0.038). One-way ANOVA with Tukey-adjusted comparisons on log_10_ vol and Games–Howell comparisons on the raw scale agreed in every pairwise contrast and are reported in full in Supplementary Table S5.

Because treatment was allocated predominantly by litter, the primary analysis was a linear mixed-effects model of log_10_ vol with a random intercept for litter. Litter accounted for a modest share of the variance (intraclass correlation 0.21), and both treatment effects persisted after accounting for it: LPS versus vehicle +1.35 log_10_ mm^3^ (95% CI 0.98 to 1.72, p < 0.001) and LPS + calpeptin versus LPS −0.61 log_10_ mm^3^ (95% CI −1.02 to −0.20, p = 0.004), the latter corresponding to a 4.0-fold reduction. Litter-mean volumes were cleanly ordered and did not overlap between vehicle (0.26, 0.30, 0.37 and 0.63 mm^3^) and LPS (3.03, 8.55 and 12.79 mm^3^), with the two calpeptin litters intermediate (1.82 and 3.47 mm^3^). Ventricular volume is therefore the outcome for which the evidence is most robust to the clustered design. Calpeptin did not normalize ventricular volume: calpeptin-treated animals remained larger than vehicle controls (mixed model +0.74 log_10_ mm^3^, 95% CI 0.36 to 1.12, p < 0.001). Two of the eight LPS animals in this analysis came from cohort 0; excluding them did not change the conclusion. All individual values are plotted in Fig. 2E and tabulated in Supplementary Table S1–S4.

### 3.5 Higher observed endpoint survival after calpeptin treatment is exploratory

Survival was analyzed as time to death (Fig. 2C–Table 1). Across all cohorts, 10 of 26 LPS-exposed animals (38.5%), 10 of 12 LPS + calpeptin animals (83.3%) and 12 of 13 vehicle animals (92.3%) survived to endpoint. Deaths were concentrated in the first days after injection: all 16 LPS deaths occurred between 1 and 4 days after injection (postnatal days 4–7), as did both calpeptin deaths, whereas the single vehicle death occurred on day 15, the scheduled endpoint day for that cohort, the animal having been found dead before endpoint imaging (Table 1). Because treatment was allocated predominantly by litter, between-litter heterogeneity was assessed before any treatment comparison, and the result is central to how these survival data should be read. Survival in the five LPS litters (Fig. 2D) was 4/5 (80.0%) in LPS 1 (the 5.0-μg litter of cohort 0), 0/5 (0%) in LPS 2 (cohort 1; 5.0 μg), 1/6 (16.7%) in LPS 3 (modulation experiment 1, cohort 2), and—within cohort 3, injected on the same day by the same operator—5/5 (100%) in LPS 4 and 0/5 (0%) in LPS 5. Between-litter heterogeneity in the LPS arm was extreme (log-rank χ^2^ = 29.36, df = 4, p = 6.6 × 10^−6^). By contrast, no heterogeneity was detectable between the four vehicle litters (χ^2^ = 0.67, p = 0.88) or the two calpeptin litters (χ^2^ = 0.03, p = 0.85). Aggregating the LPS arm to a single figure of 38.5% therefore conceals a range from complete survival to complete mortality between litters exposed to intraventricular LPS, and cohort-level analysis alone does not capture this because two of the divergent litters sit within the same cohort. One litter, in cohort 1 (Vehicle 1 and LPS 2), was divided within the litter between LPS and vehicle: all five LPS-exposed pups died while both vehicle-exposed littermates survived (Fisher exact p = 0.048). This is the only comparison in which dam, litter, injection day and operator are held constant. It provides limited within-litter evidence consistent with an LPS effect on mortality, but the small sample provides imprecise evidence and the comparison contains no calpeptin-treated animals.

The treatment comparison is reported at all three levels of analysis. At the pup level, in a Cox model stratified by cohort with vehicle as reference, LPS increased the hazard of death (HR 17.40, 95% CI 2.24 to 135.2, p = 0.0063) while LPS + calpeptin did not differ from vehicle (HR 2.86, 95% CI 0.25 to 32.0, p = 0.39); the overall likelihood-ratio test was p = 1.4 × 10^−4^. With LPS as reference, the pup-level model estimated a lower hazard in the calpeptin-treated group (HR 0.19, 95% CI 0.041 to 0.886, p = 0.035); the cluster-robust estimate was similar (HR 0.20, 95% CI 0.073 to 0.538, p = 0.0015). These analyses are exploratory: with only ten clusters, robust variance estimation can be anti-conservative, and nominal pup-level significance cannot establish a survival benefit at the litter allocation level. At the litter level, which respects the unit of allocation, mean survival was 0.92 across four vehicle litters, 0.39 across five LPS litters and 0.83 across two calpeptin litters; no comparison reached significance (Kruskal–Wallis p = 0.19; LPS versus calpeptin p = 0.43), which is unsurprising given that with five and two litters the smallest attainable p value for that comparison is 0.095. The proportional-hazards assumption was satisfied (scaled Schoenfeld residuals, all p > 0.52).

Because the number of events was small, sensitivity analyses were performed and are reported in full in Supplementary Table S5. A within-cohort permutation test of the stratified Cox statistic gave p = 0.014; unstratified pairwise log-rank tests with Holm correction and a Fisher exact test on the pooled proportions were concordant in direction and significance. As a sensitivity analysis, we also excluded the split-litter cohort: endpoint survival was then 10/21 in the remaining LPS animals versus 10/12 with LPS + calpeptin (two-sided Fisher exact p = 0.067). Taken together, the exploratory survival difference is directionally consistent but does not establish a treatment effect at the unit of allocation and is not conventionally significant after exclusion of the split-litter cohort. Pup-level and cluster-robust analyses reach conventional significance; the litter-level analysis, which is matched to the unit of allocation, does not and could not, because only two calpeptin litters were studied. Given that two confirmed 5-μg LPS litters injected on the same day differed by 100 percentage points in survival, between-litter variation remains a serious competing explanation. Establishing a survival effect in this model would require allocation of a substantially larger number of litters per arm.

### 3.6 Descriptive postnatal weight trajectories in cohort 3

Body weight was recorded serially in cohort 3, the cohort with complete measurements in all three arms. The observed trajectory of the surviving LPS litter was lower than that of the vehicle litter, whereas the trajectory of the calpeptin-treated litter was closer to the vehicle litter. At day 12 after injection, observed mean weights were 6.48 ± 0.33 g in vehicle, 5.02 ± 0.48 g in LPS and 6.23 ± 0.28 g in LPS + calpeptin animals.

These observations are descriptive. Each treatment arm consisted of one litter, treatment and litter were completely confounded, and the animals that died could not contribute later measurements. No pre-injection baseline weight was recorded. Consequently, the observed differences cannot establish a treatment effect, exclude survival bias or be interpreted as neurological or functional recovery. The individual trajectories are shown in Fig. S2.

### 3.7 Cortical vascular labeling after intraventricular LPS and calpeptin exposure

DiI vessel labeling was assessed at P17 in 16 animals from cohort 3 (vehicle n = 6, LPS n = 5 and LPS + calpeptin n = 5; 30 hemispheres analyzed). Mean hemisphere-level intensities were 63.72 ± 32.31 in vehicle, 33.61 ± 10.09 in LPS and 61.29 ± 12.41 density units in LPS + calpeptin animals. Thus, the surviving LPS litter showed lower cortical DiI labeling than the vehicle and calpeptin litters. However, each analyzed treatment group represented one litter, and the second LPS litter had no survivors. The treatment pattern is therefore descriptive and cannot be separated from litter effects or survival selection.

Labeling was modestly higher in the injected hemisphere than in the contralateral hemisphere in each group. This within-animal pattern argues against simple depletion of labeling along the ipsilateral needle track, but it does not resolve the between-litter treatment comparison. Individual hemispheres and paired measurements are shown in Fig. 3G without inferential treatment statistics.

**Fig. 3:**
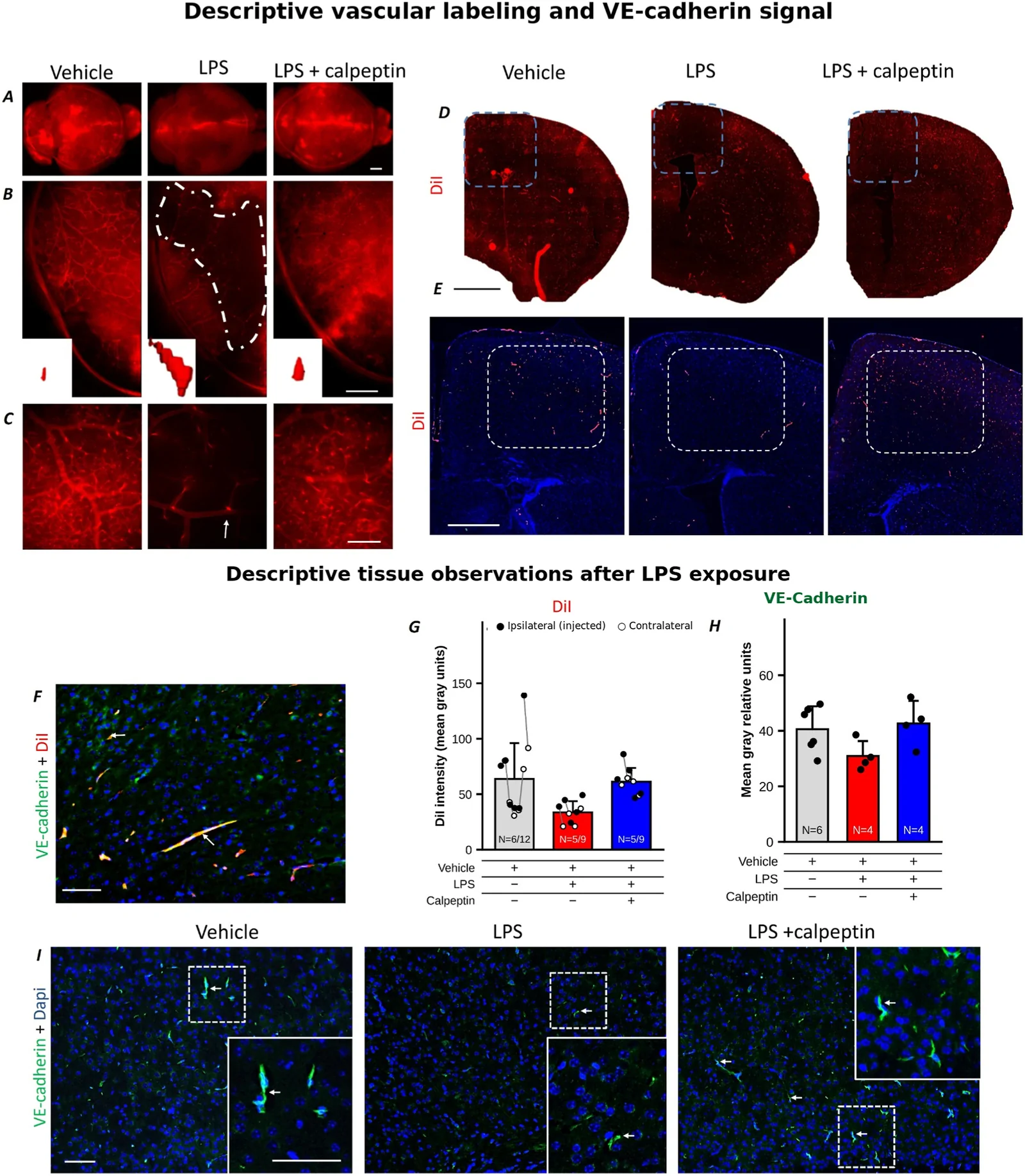
Descriptive vascular labeling and VE-cadherin signal after intraventricular LPS and calpeptin exposure. (A–F,I) Representative DiI and VE-cadherin images. Scale bars: A, B and D, 1 mm; C, F and I, 50 μm; E, 200 μm. (G) DiI intensity with hemispheres from each animal joined (vehicle n = 6 animals/12 hemispheres, LPS n = 5/9, LPS + calpeptin n = 5/9). Filled symbols denote the injected hemisphere and open symbols the contralateral hemisphere. (H) Animal-level VE-cadherin intensity (vehicle n = 6, LPS n = 4, LPS + calpeptin n = 4). All analyzed animals came from cohort 3 and represented one contributing litter per treatment group; the second LPS litter had no survivors. Treatment and litter are therefore completely confounded, and panels G and H are presented descriptively without inferential treatment comparisons. DiI is a measure of vascular labeling, and VE-cadherin immunofluorescence reports immunoreactive signal; neither endpoint directly measures blood-brain barrier permeability or demonstrates barrier protection.

Reduced DiI signal is compatible with several non-exclusive explanations, including differences in vascular density, regional perfusion at fixation, endothelial membrane condition or labeling efficiency. DiI is a vascular-labeling method rather than a barrier-integrity or permeability assay. No in vivo tracer-extravasation experiment was performed, and the cultured-cell HRP assay cannot substitute for one.

### 3.8 Descriptive in vivo VE-cadherin immunofluorescence

VE-cadherin immunofluorescence was quantified at the animal level in the cohort 3 tissue available for analysis (vehicle 40.57 ± 8.25, n = 6; LPS 30.89 ± 5.41, n = 4; LPS + calpeptin 42.67 ± 8.15 density units, n = 4). The observed group ordering was similar to that of DiI labeling.

Because each treatment group was represented by one litter, treatment and litter are completely confounded and no inferential treatment comparison is reported. These data therefore do not provide independent evidence of junctional preservation in vivo. A study with several independently allocated litters per arm, blinded quantification and direct permeability measurements would be required for such a conclusion.

Double labeling for DiI and VE-cadherin showed colocalization at cortical microvessels (Fig. 3F), confirming that the DiI-labeled structures quantified in Fig. 3G are endothelial. This is an anatomical control and does not itself demonstrate junctional integrity.

## 4 Discussion

In neonatal mice exposed to intraventricular LPS, preventive calpeptin treatment reduced mean ventricular volume by 72%, and this difference persisted after accounting for the predominantly litter-level allocation. Higher observed endpoint survival was consistent in direction across pup-level analyses but remains a descriptive, hypothesis-generating finding rather than a demonstrated treatment effect, because only two calpeptin litters were studied and LPS survival varied markedly between litters. Weight trajectories, cortical DiI labeling and in vivo VE-cadherin immunofluorescence were derived from one litter per treatment in cohort 3 and are therefore descriptive. In cultured primary brain microvascular endothelial cells, LPS increased permeability in a single experiment and increased the approximately 95/130 kDa VE-cadherin-immunoreactive band ratio across two cell preparations. Together, these observations support the possibility that calpeptin-sensitive protease activity contributes to the phenotype, but they do not identify the responsible protease, validate the lower-molecular-weight band as a cleavage product or establish an endothelial mechanism in vivo.

Calpeptin is not a calpain-selective probe and inhibits cysteine cathepsins K and L with picomolar potency [42]; no target-engagement or protease-activity assay was performed. DiI labeling and VE-cadherin immunofluorescence do not measure blood-brain barrier permeability, and no tracer-extravasation experiment was performed. The cultured-cell permeability and band-ratio responses are parallel pharmacological associations, not evidence that the band mediates permeability. Finally, LPS and calpeptin delivered into the ventricle can affect ependyma, choroid plexus epithelium, ventricular-zone progenitors, immune cells, astrocytes and pericytes as well as endothelium. Endothelial responses are therefore one candidate component of a broader inflammatory phenotype rather than an established principal mechanism.

### 4.1 Alternative and parallel mechanisms of inflammatory ventriculomegaly

Inflammatory ventricular enlargement in the neonatal brain is unlikely to arise from a single cellular compartment, and several mechanisms not assessed here could contribute to the phenotype we observed, and to the residual ventriculomegaly that persisted despite calpeptin. Fig. 4 integrates the outcomes measured in the present study with these candidate parallel pathways as a working hypothesis, without implying that causal relationships between compartments have been established.

**Fig. 4:**
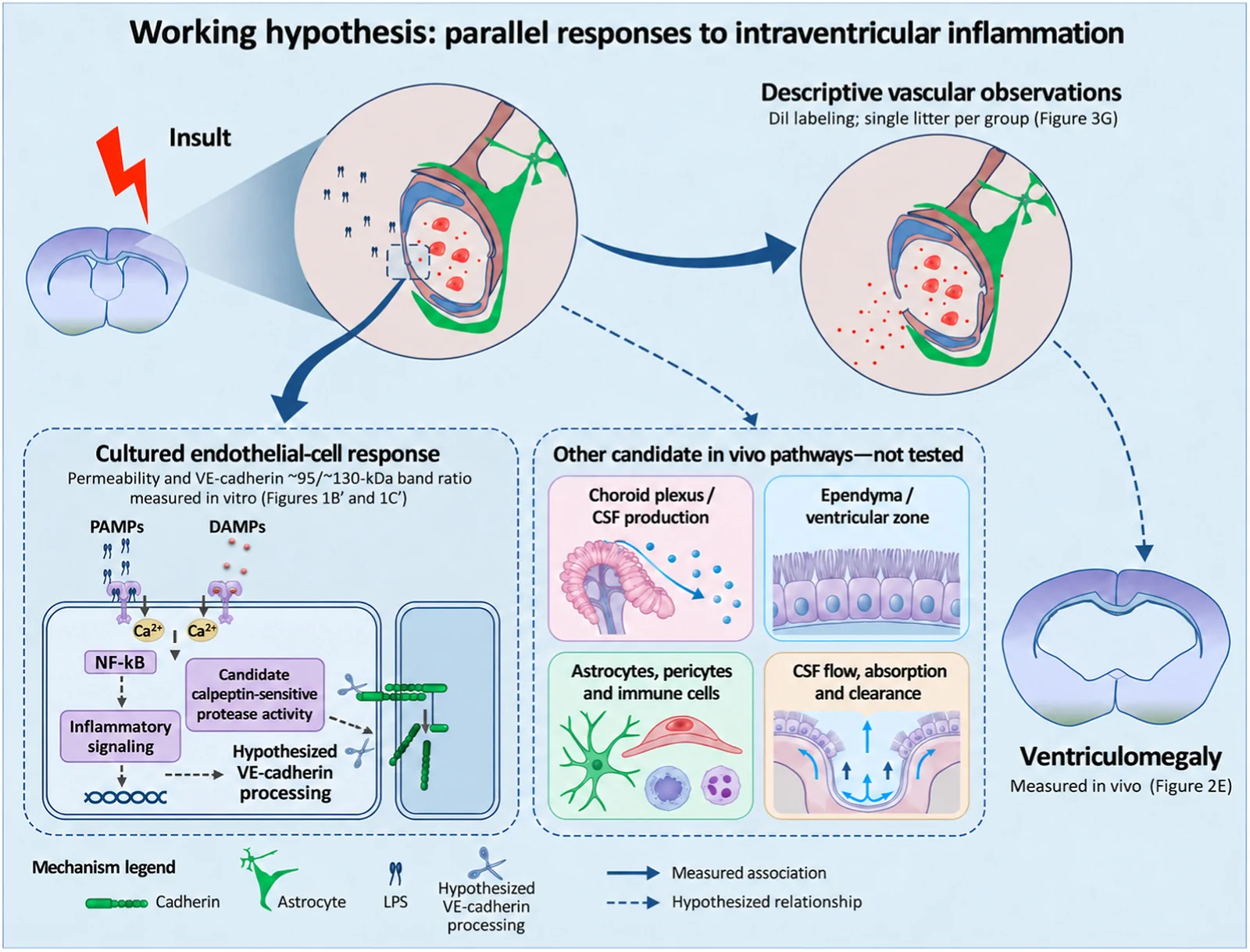
Working **hypothesis:** parallel **responses to** intraventricular **inflammation.** The schematic presents endothelial responses, choroid plexus and CSF-production changes, ependymal and ventricular-zone injury, immune/glial/pericyte responses, and altered CSF absorption or clearance as parallel candidate pathways that may contribute to ventriculomegaly. Outcomes measured in this study are cultured-cell permeability and the approximately 95/130 kDa VE-cadherin **immunoreactive** band ratio, cortical vascular labeling, and ventricular volume. **Solid blue arrows connect experimental observation domains without establishing causal mediation between the in vivo and in vitro findings. Solid arrows within the cellular schematic illustrate signaling pathways described in previous literature, including calcium and NF-κB signaling, which were not directly measured in the present study. Dashed arrows indicate hypothesized mechanistic relationships not established in these experiments. The scissors symbol denotes hypothesized VE-cadherin processing, which is a candidate mechanism rather than demonstrated cleavage of the approximately 95 kDa immunoreactive band.** Protease activation, the identity of the responsible protease, and causal links between the candidate compartments and ventricular enlargement were not **directly established.** Relative panel size reflects which outcomes were measured in this study and does not indicate the relative importance of the candidate pathways. The schematic is a working hypothesis rather than a demonstrated causal model.

Choroid plexus inflammatory remodelling and CSF hypersecretion. TLR4-dependent, SPAK/NKCC1-mediated CSF hypersecretion by the choroid plexus epithelium is an established driver of post-infectious and post-hemorrhagic hydrocephalus [12], [13], and inflammatory CSF induces proliferative and remodelling responses in choroid plexus epithelium [46]. Neither CSF production rate nor choroid plexus histology was assessed here.

Ependymal and ventricular zone injury. Junctional disruption of the ventricular zone with loss of N-cadherin and ependymal denudation is a well-characterized route to ventriculomegaly [18], [19], [20], [22], [23]. We did not examine the ependymal lining, and intraventricular delivery places LPS in direct contact with it.

Immune cell infiltration and cytokine signalling. Classical TLR4 downstream mediators (TNF-α, IL-1β, IL-6), NF-κB activation and intracellular calcium flux were not measured in this study, in cells, CSF or tissue. Their contribution is therefore unquantified.

Pericytes and astrocytic endfeet. Neurovascular unit components other than endothelium were not examined, and calpeptin would be expected to act on them as well.

Perivascular and glymphatic fluid transport and CSF absorption. Impaired perivascular clearance and altered CSF egress routes are plausible contributors to ventricular enlargement [9], [11], [47], [48] and were not assessed.

Other proteases. Matrix metalloproteinases, ADAM-family sheddases and cathepsins may all be engaged by inflammatory stimuli in the developing brain. Of these, cysteine cathepsins are also inhibited by calpeptin [42], so calpain and cathepsin contributions cannot be separated in the present experiments.

### 4.2 Potential protease pathways and cadherin processing

Published studies show that several protease families can alter cadherin-dependent junctions, with effects that depend on stimulus, cell type, substrate and developmental state. ADAM10-dependent N-cadherin cleavage has been demonstrated in ventricular-zone disruption after hemorrhage [16]; ADAM10 proteolysis of VE-cadherin and N-cadherin also regulates endothelial permeability and cell-cell adhesion in other systems [49], [50], and defines a pathway of vascular injury in bacterial sepsis and in α-hemolysin-mediated endothelial damage [51], [52]; calpains, in turn, have been associated with VE-cadherin processing in other endothelial injury models [32], [34], [35], [36], [37], [38], [39]. The present LPS experiments do not establish that either pathway mediates ventriculomegaly. The approximately 95 kDa band is compatible with protein processing but was not biochemically identified.

No ADAM10 inhibitor, metalloprotease inhibitor, calpain- or cathepsin-activity assay, isoform-specific manipulation or cleavage-resistant cadherin construct was used. We therefore interpret the data as pharmacological sensitivity to calpeptin rather than evidence for a specified calpain pathway, and direct comparison of candidate proteases remains a future experiment.

### 4.3 Why calpeptin did not normalize ventricular volume

Calpeptin-treated animals remained ventriculomegalic relative to vehicle controls (mixed model +0.74 log_10_ mm^3^, 95% CI 0.36 to 1.12, p < 0.001). Several non-exclusive explanations are consistent with our data: parallel injury pathways that are not cysteine protease–dependent (cytokine signalling, complement activation, metalloprotease activity); incomplete or short-lived target engagement, which we did not measure; injury to CSF-regulatory structures such as the choroid plexus and ependyma that had already occurred before the inhibitor reached an effective concentration; and irreversible structural change occurring within the first hours after injection. Partial attenuation is compatible with protease-sensitive activity being one contributor among several, but the present data neither identify the responsible protease nor establish a single linear TLR4 → calpain → VE-cadherin → hydrocephalus mechanism. Notably, inhibition of a single metalloproteinase in the ADAM10 arm of this framework produced near-complete normalization of ventricular volume after a hemorrhagic insult [16], whereas calpeptin treatment here produced only partial attenuation, which is consistent with distinct proteases carrying different fractions of the injury depending on the insult and the compartment engaged.

### 4.4 Translational considerations

The distance between this experiment and any clinical application is substantial, and we set out the specific barriers rather than gesture at them.

Administration was preventive, not therapeutic: calpeptin was given before and simultaneously with the insult. No therapeutic window was defined, and no rescue after established injury was attempted.

A single dose was tested in vivo. No dose–response relationship for calpeptin was established, and neither a minimum effective dose nor a toxic threshold is known in this model.

No pharmacokinetic data, brain exposure measurement or target engagement assay was obtained.

The compound lacks isoform selectivity and inhibits cathepsins with picomolar potency [42]; sustained cathepsin inhibition carries a plausible risk of lysosomal and immune toxicity in a developing organism, and calpeptin at higher doses has produced leukopenia in adult rodents [42].

The vehicle contained DMSO, which is not neutral in the developing brain: intraventricular DMSO alone induces ventriculomegaly in neonatal mice in a dose-dependent manner, with ventricular lining disruption and periventricular apoptosis [53].

Intraventricular delivery of a protease inhibitor with LPS is not a clinically deliverable regimen.

A severe intraventricular endotoxin challenge does not correspond to any single clinical etiology; post-hemorrhagic, post-infectious and post-traumatic hydrocephalus differ in their inflammatory profiles.

Only ventricular volume, weight and survival were assessed. No long-term behavioral, cognitive or neurodevelopmental outcomes were measured, and relevance to preterm human infants remains unestablished.

### 4.5 What the litter-level design does and does not permit

Allocation in this study was predominantly by litter rather than by pup, which has two consequences that we consider more important than any individual p value. First, the effective sample size for treatment comparisons is closer to the ten litters contributing to the pooled comparisons than to the fifty-one pups, so pup-level intervals are narrower than the design warrants. Second, and more concretely, the LPS arm contained two litters injected on the same day, in the same experiment, by the same operator, in which every pup of one litter survived and every pup of the other died. A between-litter difference of that magnitude is larger than the treatment effect we are attempting to detect on survival. We cannot identify its cause from the available records: candidate explanations include differences in dam care and nursing after the procedure, drift in injection depth or delivery within a single session, and undocumented differences in litter size or pup vigour at baseline.

The single within-litter contrast available (cohort 1, Section 3.5) provides limited reassurance that LPS itself affects mortality, but says nothing about calpeptin, because no litter was divided between LPS and LPS + calpeptin. The clustering does not undermine the ventricular findings, which survive a mixed model with a random intercept for litter and in which litter-mean volumes separate cleanly between vehicle and LPS litters with no overlap. It does mean that the mortality result attributed to calpeptin should be read as hypothesis-generating, and that a design allocating several litters per arm, or dividing each litter across all three treatments, would be required to establish a survival effect.

### 4.6 Limitations

Beyond the interpretive limits described above, the following methodological limitations apply.

Survivor bias in the imaging analysis. Ventricular volumes were obtained only from animals alive at endpoint MRI. Because mortality was highest in the LPS group, the LPS volumes describe a surviving subset and may underestimate the severity of injury in that group; the surviving animals should not be treated as representative of all allocated animals. Survival and ventricular volume are reported as separate outcomes and no analysis attempts to reconstruct the ventricular volumes of animals that died. Allocated, surviving, imaged and analyzed numbers are given for every group in Table 1.

Treatment was allocated predominantly by litter, not by pup. Four, five and two litters contributed to the pooled vehicle, LPS and LPS + calpeptin survival groups, respectively, and only one litter was split between treatments. Ventricular volume remains significant in a litter-adjusted model. By contrast, cohort 3 body weight, DiI labeling and VE-cadherin immunofluorescence each included only one contributing litter per treatment group, so treatment and litter cannot be separated for those endpoints and they are reported descriptively.

Extreme between-litter variation in the LPS arm. Two LPS litters injected on the same day within the same experiment showed 100% and 0% survival, and across the five LPS litters survival ranged from 0% to 100%. Whatever produced this — dam behavior, drift in injection technique within a session, or undocumented litter-level factors — it is large enough to generate a mortality difference comparable to the observed pooled contrast, and it was not controlled.

Small group sizes and no a priori power calculation. Several comparisons, in particular the in vivo VE-cadherin quantification, are underpowered, and the corresponding confidence intervals are wide.

Pooling across cohorts. The LPS arm pooled four cohorts and the vehicle arm three. Cohort-specific and litter-specific counts are reported alongside the pooled estimates. The Cox survival models were stratified by cohort; the ventricular-volume mixed-effects model included a litter random intercept, not cohort stratification.

Imaged animals are a non-random subset of survivors. Scanner availability limited cohort 0 to two scans per dose, selected in tattoo order. Ventricular volumes are therefore conditional both on survival and on selection for imaging.

No calpeptin-only control group. No animals received calpeptin without LPS, so an independent effect of calpeptin on ventricular size or growth in the absence of an inflammatory insult was not measured.

Deaths were recorded at scheduled inspections, so survival times are interval-censored to within approximately 24–48 h of the true time of death.

The cultured endothelial cells are not established as neonatal and were studied in monoculture without astrocytes or pericytes; developmental and neurovascular-unit equivalence to the P3 mouse brain was not demonstrated. The manufacturer reports endothelial identity characterization and screening for microbial contamination, but no additional authentication or mycoplasma testing was performed after receipt. The study did not include a calpain or cathepsin activity assay, isoform-specific pharmacological or genetic manipulation, cleavage-site analysis, or an in vivo permeability assay. Sex was not recorded and could not be analyzed.

### 4.7 Future directions

The experiments that would convert the present pharmacological association into a causal claim are, in our view: (i) Tlr4-knockout or endothelial-specific Tlr4-deficient animals, to establish TLR4 dependence; (ii) endothelial-specific conditional deletion of Capn1 and/or Capn2, or cleavage-resistant VE-cadherin knock-in, to establish protease and substrate specificity; (iii) in vivo tracer-extravasation assays (fluorescent dextran, Evans blue, endogenous IgG) to measure barrier permeability directly; (iv) calpeptin dose–response and delayed-administration studies with pharmacokinetic and target-engagement measurement; (v) measurement of NF-κB activation, intracellular calcium and cytokine release in cells, CSF and tissue; (vi) head-to-head comparison of selective calpain, cathepsin and ADAM10 manipulation across hemorrhagic and endotoxin insults, to test whether calpeptin-sensitive and ADAM10-dependent proteolysis act in distinct cellular compartments; and (vii) a within-litter allocated design in which each litter contributes pups to every treatment arm, which would remove between-litter variation as a competing explanation. These studies are beyond the scope of the present report.

## 5 Conclusion

Preventive calpeptin treatment attenuated LPS-induced ventriculomegaly in neonatal mice, whereas the higher observed endpoint survival remained exploratory and does not establish a survival benefit because of predominantly litter-level allocation, marked between-litter variation and only two calpeptin-treated litters. The weight and tissue-labeling observations from cohort 3 were descriptive because each treatment group came from one litter. In cultured primary brain microvascular endothelial cells, LPS increased permeability and the approximately 95/130 kDa VE-cadherin-immunoreactive band ratio, the band-ratio increase was reduced by calpeptin and by TLR4 inhibition, whereas the permeability increase was not significantly attenuated by either agent. Because calpeptin is neither protease- nor isoform-selective and direct target engagement was not measured, these findings only support a possible contribution of calpeptin-sensitive protease activity to inflammatory ventriculomegaly. They do not identify a specific protease, establish that the lower-molecular-weight immunoreactive band is a cleavage product, demonstrate blood-brain barrier protection, or establish a causal endothelial-junction mechanism in vivo.

## Ethics declaration

This study was conducted in accordance with the ARRIVE (Animal Research: Reporting of In Vivo Experiments) guidelines. This study was approved by the Institutional Animal Care and Use Committee (IACUC) of CHOC Children's Hospital/CHOC Children's Research Institute, Orange, California, USA — protocol IACUC-24-004, initial approval 13 August 2021, previously registered as protocol 210679. This covers the animal work. University of California, Irvine IACUC — protocol AUP 20-172, covering the MRI performed at the UC Irvine Preclinical and Translational Neuroimaging Center.

## Declaration of generative AI and AI-assisted technologies in the manuscript preparation process

While preparing this work, the authors used Grammarly and large language model–based assistants (ChatGPT and Claude) to improve the language and readability of the manuscript and to assist with statistical re-analysis code for the survival, body-weight and ventricular-volume data. After using these tools, the authors reviewed and edited the content as needed and take full responsibility for the publication's content.

## Funding

This work was supported by the Office of the Assistant Secretary of Defense for Health Affairs through the Peer Reviewed Medical Research Program under Award No. HT9425-25-1-0112, and by the 10.13039/100001034CHOC Foundation under Award No. CSO87, both to Leandro Castañeyra-Ruiz. Opinions, interpretations, conclusions and recommendations are those of the authors and are not necessarily endorsed by the Department of Defense.

## Declaration of competing interest

All authors’ institutional affiliations are listed on the title page of the manuscript.

The authors and their immediate family members have no financial interests, advisory roles, consulting positions, or related patent applications to declare.

The authors declare no competing interests.

All funding sources for this study are listed in the Funding section of the manuscript.

On behalf of all authors, I confirm that all relevant competing interests related to this work have been disclosed.

## Data Availability

All data supporting the findings of this study are included within the article and its supplementary information. Individual animal-level data for every in vivo experiment — animal identification, date of birth, date of injection, treatment, serial body weights, mortality and ventricular volumes — are provided in Supplementary Table S1–S4. Original uncropped Western blot membranes and permeability assay images corresponding to Fig. 1 are provided in the Supplementary Material. Analysis code used for the survival, body-weight and ventricular-volume analyses is available from the Lead Contact on request. No custom software was generated. Further information and requests for resources and reagents should be directed to the Lead Contact, Leandro Castañeyra-Ruiz (Leandro.Castaneyra.Ruiz@choc.org). This study did not generate new unique reagents or new animal lines.
