# Supplementary material for: Calpeptin attenuates LPS-induced ventriculomegaly in neonatal mice
Source: Biochem Biophys Rep. 2026 Nov 10;48:102890. doi: 10.1016/j.bbrep.2026.102890 (PMC13770645; doi:10.1016/j.bbrep.2026.102890)
Supplement: Supplementary file 1 [file mmc1.docx]

**Figure S1. Dose-selection feasibility experiment for intraventricular LPS.**

**(A)** Schematic overview of the experimental design for intraventricular LPS injection in neonatal mice.

**(B)** Representative MRI images of the lateral ventricles in animals injected with 2.5, 5.0 or 7.5 μg of LPS.

**(C)** Kaplan–Meier survival after intraventricular LPS at each dose (n = 5 pups per dose). Tick marks indicate censoring at the endpoint. Endpoint survival was 5/5 at 2.5 μg, 4/5 at 5.0 μg and 2/5 at 7.5 μg; all deaths occurred within 2 days of injection.

**(D)** Ventricular volume at endpoint MRI in the imaged survivors (n = 2 per dose), showing every individual observation; bars indicate the mean of the two animals.

This experiment was performed to select a dose for the subsequent pharmacological experiments and was not powered to establish a quantitative dose–response relationship; no correlation statistic is reported. Each dose was administered to a single litter, so dose and litter are completely confounded. Individual animal records are given in Supplementary Table S1. Scale bar, 1 mm.
