# Supplementary material for: Calpeptin attenuates LPS-induced ventriculomegaly in neonatal mice
Source: Biochem Biophys Rep. 2026 Nov 10;48:102890. doi: 10.1016/j.bbrep.2026.102890 (PMC13770645; doi:10.1016/j.bbrep.2026.102890)
Supplement: Supplementary file 2 [file mmc2.docx]

**Figure S2.** Descriptive body weight trajectories after intraventricular LPS with and without calpeptin. Serial body weights of surviving animals in cohort 3 (vehicle n = 6, LPS n = 5, LPS + calpeptin n = 6) are plotted as group means ± SD. No weights were recorded on day 2, and only three animals per group were weighed on day 13; day 12 is used for the descriptive cross-sectional summary in the Results. Each treatment arm comprised one litter, so treatment and litter are completely confounded. Because no pre-injection baseline weights were recorded and animals that died could not contribute later measurements, no inferential treatment comparison is presented; weight trajectories do not establish functional recovery.
