# Supplementary material for: Calpeptin attenuates LPS-induced ventriculomegaly in neonatal mice
Source: Biochem Biophys Rep. 2026 Nov 10;48:102890. doi: 10.1016/j.bbrep.2026.102890 (PMC13770645; doi:10.1016/j.bbrep.2026.102890)
Supplement: Supplementary file 3 [file mmc3.docx]

| **Pilot LPS Dose-Finding Experiment** | | | | | | | | | | | | | | |
| --- | --- | --- | --- | --- | --- | --- | --- | --- | --- | --- | --- | --- | --- | --- |
|  |  |  |  | **12/22/21** | **12/23/21** | **12/24/21** | **12/25/21** | **12/27/21** | **12/28/21** | **12/29/21** | **1/1/22** | **1/3/22** | **1/4/22** |  |
| **DOB** | **ID** | **D I** | **LPS-dosage** | **Weight (g)** | | | | | | | | | | **V V (mm3)** |
| **Group #1 - 1 Microliters** | | | | | | | | | | | | | | |
| 12/20/2021 | A | 12/22/2021 | 2.5 µg | 1.8 | 1.6 | 1.7 | 1.9 | 2.8 | 3.3 | 3.8 | 5.4 | 6.2 | 6.68 | 5.802 |
|  | B | 12/22/2021 | 2.5 µg | 1.6 | 1.6 | 1.7 | 1.8 | 2.9 | 3.4 | 3.8 | 5.4 | 6.1 | 6.4 | 8.48 |
|  | C | 12/22/2021 | 2.5 µg | 1.6 | 1.5 | 1.6 | 1.9 | 2.8 | 3.3 | 3.8 | 5.4 | 6.1 | 6.4 | - |
|  | D | 12/22/2021 | 2.5 µg | 1.7 | 1.6 | 1.8 | 2.1 | 3.4 | 3.8 | 4.2 | 5.8 | 6.6 | - | - |
|  | AB | 12/22/2021 | 2.5 µg | 1.7 | 1.6 | 1.6 | 1.8 | 2.6 | 2.8 | 3.3 | 4.9 | 5.8 | 6.31 | - |
|  |  |  | No Tx | 1.8 | 2 | 2.1 | 2.4 | 3.6 | 4.1 | 4.5 | 6.1 | 6.7 | - | - |
| **Group #2 - 2 Microliters** | | | | | | | | | | | | | | |
| 12/20/2021 | A | 12/22/2021 | 5 µg | 1.5 | 1.4 | 1.6 | 1.8 | 2.6 | 3.1 | 3.4 | 4.8 | 5.8 | 6.41 | 14.84 |
|  | B | 12/22/2021 | 5 µg | 1.8 | 1.8 | 1.6 | 1.8 | 2.7 | 3 | 3.4 | 5 | 5.8 | 6.23 | 10.74 |
|  | C | 12/22/2021 | 5 µg | 1.6 | 1.4 | 1.7 | 2 | 3.1 | 3.6 | 4 | 5.8 | 6.9 | 6.97 | - |
|  | D | 12/22/2021 | 5 µg | 1.5 | 1.4 | 1.4 | 1.6 | 2.6 | 3 | 3.3 | 4.7 | 5.7 | 6 | - |
|  | AB | 12/22/2021 | 5 µg | 1.5 | 1.5 | FD | X | X | X | X | X | X | X | X |
|  |  |  | No Tx | 1.6 | 1.9 | 2.1 | 2.3 | 3.3 | 3.9 | 4.4 | 5.6 | 6.7 | - | - |
|  |  |  | No Tx | 1.7 | 2 | 2.4 | 2.6 | 3.8 | 4.5 | 4.9 | 6.5 | 7.4 | - | - |
| **Group #3 - 3 Microliters** | | | | | | | | | | | | | | |
| 12/20/2021 | A | 12/22/2021 | 7.5 µg | 1.3 | 1.2 | FD | X | X | X | X | X | X | X | X |
|  | B | 12/22/2021 | 7.5 µg | 1.4 | FD | X | X | X | X | X | X | X | X | X |
|  | C | 12/22/2021 | 7.5 µg | 1.3 | 1.2 | FD | X | X | X | X | X | X | X | X |
|  | D | 12/22/2021 | 7.5 µg | 1.3 | 1.3 | 1.4 | 1.7 | 2.6 | 3 | 3.5 | 5.2 | 6.2 | 6.48 | 24.74 |
|  | AB | 12/22/2021 | 7.5 µg | 1.4 | 1.3 | 1.4 | 1.7 | 2.7 | - | 3.6 | 5.2 | 6.1 | 6.49 | 22.13 |
|  |  |  | No Tx | 1.3 | 1.6 | 2 | 2.3 | 3.5 | 3.9 | 4.7 | 6.2 | 7.3 | - |  |
|  |  |  | No Tx | 1.4 | 1.8 | 2.2 | 2.7 | 3.9 | 4.5 | 5.2 | 7 | 7.8 | - |  |
|  |  |  | No Tx | 1.4 | 1.8 | 2.2 | 2.5 | 3.8 | 4.3 | 4.9 | 6.6 | 7.7 | - |  |

**Table S1. Dose-response experimental data (cohort 0).** The table indicates the day of birth (DOB), identification (ID), date of injection (DI) LPS Dosage, Weights and ventricular volumes (V V). FD, Found deceased. No Tx, No treated. X, No data. (-), No imaged. Entries recorded after the date of death are shown as X. No Tx animals were uninjected littermates maintained as a growth reference and were not imaged. Only the 5.0 µg litter (litter B) contributes to the pooled LPS arm, where it is designated LPS 1 in Figures 2D and 2E; the 2.5 µg and 7.5 µg litters are not part of that arm and are not numbered.

| **Experiment 0 — LPS and vehicle within a single litter (cohort 1)** | | | | | | |
| --- | --- | --- | --- | --- | --- | --- |
| **DOB** | **ID** | **D I** | **Treatment** | **Outcome** | **Day post-injection** | **V V (mm³)** |
| **LPS (5 µg)** | | | | | | |
| 2/7/2022 | A | 2/9/2022 | LPS | FD | 2 | X |
|  | B | 2/9/2022 | LPS | FD | 2 | X |
|  | C | 2/9/2022 | LPS | FD | 2 | X |
|  | D | 2/9/2022 | LPS | FD | 2 | X |
|  | AC | 2/9/2022 | LPS | FD | 2 | X |
| **Vehicle (2 µl of vehicle)** | | | | | | |
|  | AB | 2/9/2022 | Vehicle | Survived | 14 | 0.295 |
|  | AD | 2/9/2022 | Vehicle | Survived | 14 | 0.450 |

**Table S2. Experiment 0 — LPS and vehicle within a single litter.** This litter is the only one divided within the litter between treatments and provides the sole within-litter contrast in the study (cohort 1 in the manuscript). Its two arms are designated Vehicle 1 and LPS 2 in Figures 2D and 2E. Five pups received intraventricular LPS and two littermates received vehicle on the same day, by the same operator, from the same dam. Body weights were not recorded for this cohort. The LPS-exposed pups received the 5 µg intraventricular dose used in all experiments other than the dose-selection cohort. The table indicates the day of birth (DOB), identification (ID), date of injection (DI), treatment, outcome, day post-injection at death or last observation, and ventricular volumes (V V). FD, Found deceased. X, No data.

|  | **Calpain modulation experiment 1.** | | | | | | | | | | | |
| --- | --- | --- | --- | --- | --- | --- | --- | --- | --- | --- | --- | --- |
|  |  |  |  |  | **4/22/2022** | **4/25/2022** | **4/27/2022** | **4/30/2022** | **5/2/2022** | **5/4/2022** | **5/6/2022** |  |
|  | **DOB** | **ID** | **DI** | **Treatment** | **Weight (g)** | | | | | | | **V V (mm^3^)** |
|  | **Vehicle 2 — control #1 (2 µl of vehicle)** | | | | | | | | | | | |
|  | 4/18/2022 | A | 4/21/2022 | Vehicle | 1.1 | 1.8 | 2.3 | 3.2 | 3.4 | 2.5 | FD | X |
|  |  | B | 4/21/2022 | Vehicle | 1.3 | 2.3 | 3.2 | 4.4 | 4.7 | 4.4 | 3.4 | 0.07 |
|  |  | C | 4/21/2022 | Vehicle | 1.7 | 3 | 3.9 | 5 | 5.2 | 5 | 4.2 | 0.49 |
|  | **Vehicle 3 — control #2 (2 µl of vehicle)** | | | | | | | | | | | |
|  | 4/18/2022 | A | 4/21/2022 | Vehicle | 2.6 | 4.8 | 5.9 | 8.1 | 8.6 | 8.9 | 8.7 | 0.34 |
|  |  | B | 4/21/2022 | Vehicle | 2.5 | 4.5 | 5.9 | 7.9 | 8.1 | 8.5 | 8.48 | 0.26 |
|  | **LPS (5 µg in 2 µl of vehicle)** | | | | | | | | | | | |
|  | 4/18/2022 | A | 4/21/2022 | LPS | 1.5 | FD | X | X | X | X | X | X |
|  |  | B | 4/21/2022 | LPS | 1.5 | 1.8 | 2 | 3.4 | 3.6 | 3.8 | 3.6 | 3.03 |
|  |  | C | 4/21/2022 | LPS | 1.7 | FD | X | X | X | X | X | X |
|  |  | D | 4/21/2022 | LPS | 1.4 | FD | X | X | X | X | X | X |
|  |  | AB | 4/21/2022 | LPS | 1.6 | FD | X | X | X | X | X | X |
|  |  | AC | 4/21/2022 | LPS | 1.5 | FD | X | X | X | X | X | X |
|  | **LPS + Calpeptin (5 µg of LPS and 15 μg of calpeptin in 2 µl of vehicle)** | | | | | | | | | | | |
|  | 4/18/2022 | A | 4/21/2022 | LPS+Calp | 1.5 | 1.9 | 3.1 | 4.7 | 5.1 | 5.1 | 5.3 | 6.279 |
|  |  | B | 4/21/2022 | LPS+Calp | 1.7 | 2.1 | 3.2 | 4.9 | 4.8 | 4.8 | 5.2 | 2.19 |
|  |  | C | 4/21/2022 | LPS+Calp | 1.5 | 2.1 | 3.3 | 5 | 5.3 | 5.7 | 5.8 | 3.657 |
|  |  | D | 4/21/2022 | LPS+Calp | 1.4 | 1.6 | 2.4 | 4.1 | 4.3 | 4.3 | 4.8 | - |
|  |  | AB | 4/21/2022 | LPS+Calp | 1.3 | FD | X | X | X | X | X | X |

**Table S3.** Calpain modulation experimental data 1 (cohort 2). Litters in this cohort are designated Vehicle 2 (control #1), Vehicle 3 (control #2), LPS 3 and Calpeptin 1 in Figures 2D and 2E. The table indicates the day of birth (DOB), identification (ID), date of injection (DI), treatment, weights and ventricular volumes (V V). FD, Found deceased. X, No data.

| **Calpain modulation experiment 2** | | | | | | | | | | | | | |
| --- | --- | --- | --- | --- | --- | --- | --- | --- | --- | --- | --- | --- | --- |
|  |  |  |  | **6/4/2022** | **6/5/2022** | **6/6/2022** | **6/8/2022** | **6/10/2022** | **6/12/2022** | **6/14/2022** | **6/15/2022** | **6/16/2022** |  |
| **DOB** | **Tattoo** | **DI** | **Treatment** | **Weight (g)** | | | | | | | | | **V V ( mm^3^)** |
| **Vehicle 4 — control (2 µl of vehicle)** | | | | | | | | | | | | | |
| 5/31/2022 | A | 6/3/2022 | Vehicle | 1.8 | - | 2.6 | 3.8 | 4.3 | 5.2 | 5.9 | 6.1 | N/A | 0.52 |
|  | B | 6/3/2022 | Vehicle | 2 | - | 2.7 | 3.7 | 4.65 | 5.4 | 6.2 | 6.5 | N/A | 0.55 |
|  | C | 6/3/2022 | Vehicle | 2 | - | 2.9 | 3.85 | 4.7 | 5.7 | 6.6 | 6.9 | N/A | 1.53 |
|  | D | 6/3/2022 | Vehicle | 1.9 | - | 2.6 | 3.85 | 4.4 | 5.5 | 6.3 | 6.6 | 7.5 | 0.38 |
|  | AB | 6/3/2022 | Vehicle | 2.1 | - | 3 | 3.9 | 4.8 | 5.9 | 6.6 | 6.7 | 7.4 | 0.45 |
|  | NM | 6/3/2022 | Vehicle | 2 | - | 2.8 | 3.7 | 4.25 | 5 | 5.8 | 6.1 | 6.7 | 0.27 |
| **LPS + Calpeptin (5 µg of LPS and 15 μg of calpeptin in 2 µl of vehicle)** | | | | | | | | | | | | | |
| 5/31/2022 | A | 6/3/2022 | LPS+Calp | 1.7 | - | 2.2 | 3.3 | 4.15 | 5.4 | 6.2 | 6.6 | N/A | 1.12 |
|  | B | 6/3/2022 | LPS+Calp | 1.6 | - | 2.1 | 3 | 3.8 | 5.1 | 6 | 6.3 | N/A | 0.24 |
|  | C | 6/3/2022 | LPS+Calp | 1.6 | - | 2.1 | 3.3 | 4.05 | 5.1 | 6.1 | 6.4 | N/A | 0.97 |
|  | D | 6/3/2022 | LPS+Calp | 1.6 | - | 1.8 | 2.75 | 3.55 | 4.9 | 5.6 | 5.9 | 6.6 | 4.36 |
|  | AB | 6/3/2022 | LPS+Calp | 1.6 | - | 1.8 | 2.95 | 3.85 | 5 | 5.9 | 6.3 | 7 | 2.82 |
|  | AC | 6/3/2022 | LPS+Calp | 1.7 | - | 2 | 2.9 | 3.8 | 4.8 | 5.5 | 5.9 | 6.5 | 1.4 |
|  | NM | 6/3/2022 | LPS+Calp | FD | X | X | X | X | X | X | X | X | X |
| **LPS (5 µg in 2 µl of vehicle)** | | | | | | | | | | | | | |
| 5/31/2022 | A | 6/3/2022 | LPS | 1.8 | - | 2.1 | 3.1 | 4.2 | 4.6 | 5.2 | 5.6 | N/A | 9.92 |
|  | B | 6/3/2022 | LPS | 1.6 | - | 1.7 | 2.4 | 3.1 | 3.5 | 4 | 4.4 | N/A | 7.22 |
|  | C | 6/3/2022 | LPS | 1.6 | - | 1.8 | 2.8 | 3.65 | 4 | 4.4 | 4.8 | 5.2 | 6.86 |
|  | D | 6/3/2022 | LPS | 1.8 | - | 2 | 2.95 | 3.55 | 4 | 4.5 | 4.9 | 5.4 | 5.13 |
|  | NM | 6/3/2022 | LPS | 1.7 | - | 2 | 3.1 | 3.9 | 4.5 | 5.1 | 5.4 | 6.1 | 13.61 |
| **LPS (5 µg in 2 µl of vehicle)** | | | | | | | | | | | | | |
| 5/31/2022 | A | 6/3/2022 | LPS | 1.5 | FD | X | X | X | X | X | X | X | X |
|  | B | 6/3/2022 | LPS | 1.6 | FD | X | X | X | X | X | X | X | X |
|  | C | 6/3/2022 | LPS | FD | X | X | X | X | X | X | X | X | X |
|  | D | 6/3/2022 | LPS | FD | X | X | X | X | X | X | X | X | X |
|  | NM | 6/3/2022 | LPS | FD | X | X | X | X | X | X | X | X | X |

**Table S4**. Calpain modulation experimental data 2 (cohort 3). Litters in this cohort are designated Vehicle 4, LPS 4, LPS 5 and Calpeptin 2 in Figures 2D and 2E. The table indicates the day of birth (DOB), identification (ID), date of injection (DI), treatment, weights and ventricular volumes (V V). FD, Found deceased. X, No data.

**Supplementary Table S5. Complete statistical reporting for all in vivo and immunofluorescence outcomes.**

*Primary analyses are indicated; all remaining entries are sensitivity or secondary analyses reported here in full so that no test performed is omitted from the record. Analyses were performed in GraphPad Prism 10 and Python 3.12 (lifelines 0.30.3, statsmodels, SciPy).*

| **Outcome** | **Analysis** | **Comparison** | **Result** |
| --- | --- | --- | --- |
| Endothelial permeability (Figure 1B′) | Kruskal–Wallis, retained wells (primary) | Omnibus, 22 wells | H = 12.59, p = 0.0056 |
|  | Dunn–Holm, retained wells | LPS vs vehicle | p = 0.029 |
|  |  | LPS + calpeptin vs LPS | p = 1.00 |
|  |  | LPS + TLR4 inhibitor vs LPS | p = 1.00 |
|  |  | LPS + calpeptin vs vehicle | p = 0.058 |
|  |  | LPS + TLR4 inhibitor vs vehicle | p = 0.010 |
|  | Sensitivity analysis: all wells retained | Omnibus, 26 wells (including the four exceeding the cell-free control) | H = 6.44, p = 0.092; no pairwise comparison significant after Holm correction |
|  |  | LPS vs vehicle, all 26 wells | Dunn–Holm p = 0.090 (unadjusted Mann–Whitney p = 0.043) |
| VE-cadherin 95/130 kDa immunoreactive band ratio (Figure 1C′) | ANOVA on log₁₀ ratio, experiment as blocking factor (primary) | Group effect | F(3,29) = 24.86, p < 0.0001 |
|  |  | Block (experiment) effect | p = 0.0003 |
|  | Tukey-adjusted post hoc | LPS vs vehicle | p = 0.0001 |
|  |  | LPS + calpeptin vs LPS | p < 0.0001 |
|  |  | LPS + TLR4 inhibitor vs LPS | p = 0.0001 |
|  |  | LPS + calpeptin vs vehicle | p = 0.0055 |
|  |  | LPS + TLR4 inhibitor vs vehicle | p = 0.98 |
|  |  | LPS + calpeptin vs LPS + TLR4 inhibitor | p = 0.025 |
|  | Within-experiment permutation test | Group effect | p = 0.0001 (10,000 permutations) |
|  | Kruskal–Wallis within each experiment | Experiment 1 / experiment 2 | p = 0.0057 / p = 0.0015 |
| Ventricular volume | One-way ANOVA, log₁₀ volumes | Omnibus | F(2,27) = 40.08, p = 8.3 × 10⁻⁹ |
|  | Tukey-adjusted post hoc, log₁₀ | LPS vs vehicle | p < 0.0001 |
|  |  | LPS + calpeptin vs LPS | p = 0.0008 |
|  |  | LPS + calpeptin vs vehicle | p = 0.0001 |
|  | Games–Howell, raw scale | LPS vs vehicle | p = 0.0016 |
|  |  | LPS + calpeptin vs LPS | p = 0.0062 |
|  |  | LPS + calpeptin vs vehicle | p = 0.018 |
|  | Kruskal–Wallis with Dunn–Holm | Omnibus | H = 21.01, p = 2.7 × 10⁻⁵ |
|  |  | Pairwise (as above order) | p = 2.0 × 10⁻⁵, 0.038, 0.038 |
|  | Linear mixed-effects model (primary), random intercept for litter | LPS vs vehicle | +1.35 log₁₀ mm³ (95% CI 0.98 to 1.72), p < 0.001 |
|  |  | LPS + calpeptin vs LPS | −0.61 log₁₀ mm³ (95% CI −1.02 to −0.20), p = 0.004 |
|  |  | LPS + calpeptin vs vehicle | +0.74 log₁₀ mm³ (95% CI 0.36 to 1.12), p < 0.001 |
|  | Intraclass correlation | Litter | ICC = 0.21 |
| Survival | Between-litter heterogeneity, log-rank | LPS arm (5 litters) | χ² = 29.36, df = 4, p = 6.6 × 10⁻⁶ |
|  |  | Vehicle arm (4 litters) | χ² = 0.67, p = 0.88 |
|  |  | Calpeptin arm (2 litters) | χ² = 0.03, p = 0.85 |
|  | Cox model stratified by cohort (primary) | LPS vs vehicle | HR 17.40 (95% CI 2.24–135.2), p = 0.0063 |
|  |  | LPS + calpeptin vs vehicle | HR 2.86 (95% CI 0.25–32.0), p = 0.39 |
|  |  | LPS + calpeptin vs LPS | HR 0.19 (95% CI 0.041–0.886), p = 0.035 |
|  | Cox model, cluster-robust SE by litter | LPS + calpeptin vs LPS | HR 0.20 (95% CI 0.073–0.538), p = 0.0015 (10 clusters; anti-conservative) |
|  | Litter-level analysis (unit of allocation) | Omnibus (Kruskal–Wallis) | p = 0.19 |
|  |  | LPS vs LPS + calpeptin | p = 0.43 (minimum attainable p = 0.095) |
|  | Within-cohort permutation test (2000 permutations) | Stratified Cox z statistic | p = 0.014 |
|  | Unstratified pairwise log-rank, Holm-corrected | LPS vs vehicle | p = 0.0009 (Holm p = 0.0027) |
|  |  | LPS vs LPS + calpeptin | p = 0.016 (Holm p = 0.033) |
|  | Fisher exact test, pooled proportions | LPS vs LPS + calpeptin | p = 0.017 |
|  | Fisher exact test, within-litter (cohort 1) | LPS vs vehicle littermates | p = 0.048 (5 vs 2 pups) |
|  | Proportional-hazards check | Scaled Schoenfeld residuals | All p > 0.52 |
| VE-cadherin immunofluorescence | One-way ANOVA (animal-level means) | Omnibus | F(2,11) = 2.86, p = 0.100 |
|  | Tukey-adjusted post hoc | LPS vs vehicle | −9.68 (95% CI −22.85 to +3.49), p = 0.16, g = −1.19 |
|  |  | LPS + calpeptin vs LPS | +11.78 (95% CI −2.65 to +26.20), p = 0.11, g = +1.48 |
|  |  | LPS + calpeptin vs vehicle | +2.10 (95% CI −11.07 to +15.27), p = 0.90, g = +0.23 |
| DiI vessel labeling | Linear mixed-effects model, random intercept for animal | Vehicle vs LPS | +0.248 log₁₀ (95% CI 0.082 to 0.413), p = 0.003 |
|  |  | LPS + calpeptin vs LPS | +0.269 log₁₀ (95% CI 0.094 to 0.445), p = 0.003 |
|  |  | Hemisphere side and interaction | side not significant; group × side p = 0.45; ICC (animal) = 0.41 |
| Body weight (cohort 3, descriptive) | Linear mixed-effects model | LPS × day vs vehicle | −0.104 g/day (95% CI −0.143 to −0.064), p < 0.001 |
|  |  | Calpeptin × day vs vehicle | +0.028 g/day (95% CI −0.010 to 0.066), p = 0.15 |
|  | One-way ANOVA, day 12 | Omnibus | F(2,14) = 24.6, p < 0.0001 |

Cluster-robust inference with only ten clusters is anti-conservative and the corresponding intervals should not be read as more secure than the pup-level estimates. The litter-level analysis is the comparison matched to the unit of allocation.

**Uncropped membranes from Figure 1C. WB 1 (negative and positive)**

Uncropped Western blot images corresponding to Figure 1C. WB1 (Experiment 1) was performed using a single membrane containing all experimental conditions.

**Uncropped membrane. WB 2 (2 membranes)**

WB2 (Experiment 2) was performed using two membranes due to sample loading constraints, with conditions distributed across membranes as shown. No lanes were rearranged within membranes, and all samples were processed in parallel under identical experimental conditions.

**Uncropped permeability experiment from Figure 1B**

LPS-TLR4-INH

LPS-calpeptin

LPS

Vehicle

No HRP

No cells
